# Root ethylene mediates rhizosphere microbial community reconstruction when chemically detecting cyanide produced by neighbouring plants

**DOI:** 10.1186/s40168-019-0775-6

**Published:** 2020-01-18

**Authors:** Yan Chen, Michael Bonkowski, Yi Shen, Bryan S. Griffiths, Yuji Jiang, Xiaoyue Wang, Bo Sun

**Affiliations:** 1 State Key Laboratory of Soil and Sustainable Agriculture, Institute of Soil Science, Chinese Academy of Sciences, No.71 East Beijing Road, Nanjing, 210008 China; 2 Terrestrial Ecology, Institute of Zoology, University of Cologne, Zülpicher Str 47b, 50674 Cologne, Germany; 3 Cluster of Excellence on Plant Sciences (CEPLAS), University of Cologne, Cologne, Germany; 4 Institute of Industrial Crops, Jiangsu Academy of Agricultural Sciences, No.50 Zhonglin Street, Nanjing, 210014 China; 5 SRUC, Crop and Soil System Research Group, West Mains Road, Edinburgh, EH93JG UK

**Keywords:** Ethylene signal, Neighbour detection, Chemical cue, Interspecific interaction, Cyanide, Rhizosphere microbial assemblage, Plant fitness

## Abstract

**Background:**

Stress-induced hormones are essential for plants to modulate their microbiota and dynamically adjust to the environment. Despite the emphasis of the role of the phytohormone ethylene in the plant physiological response to heterospecific neighbour detection, less is known about how this activated signal mediates focal plant rhizosphere microbiota to enhance plant fitness. Here, using 3 years of peanut (*Arachis hypogaea* L.), a legume, and cyanide-containing cassava (*Manihot esculenta* Crantz) intercropping and peanut monocropping field, pot and hydroponic experiments in addition to exogenous ethylene application and soil incubation experiments, we found that ethylene, a cyanide-derived signal, is associated with the chemical identification of neighbouring cassava and the microbial re-assemblage in the peanut rhizosphere.

**Results:**

Ethylene production in peanut roots can be triggered by cyanide production of neighbouring cassava plants. This gaseous signal alters the microbial composition and re-assembles the microbial co-occurrence network of peanut by shifting the abundance of an actinobacterial species, *Catenulispora* sp., which becomes a keystone in the intercropped peanut rhizosphere. The re-assembled rhizosphere microbiota provide more available nutrients to peanut roots and support seed production.

**Conclusions:**

Our findings suggest that root ethylene acts as a signal with a dual role. It plays a role in perceiving biochemical cues from interspecific neighbours, and also has a regulatory function in mediating the rhizosphere microbial assembly, thereby enhancing focal plant fitness by improving seed production. This discovery provides a promising direction to develop novel intercropping strategies for targeted manipulations of the rhizosphere microbiome through phytohormone signals.

Video abstract.

## Background

Rhizosphere microorganisms are a reservoir of additional functions that extend the plant's ability to adapt to various environmental conditions and changes [1, 2]. Plants determine the rhizosphere microbiome depending on the composition of root-secreted metabolites [3, 4]. In natural environments, plants constantly adjust the composition and concentration of root metabolites in response to various kinds of biotic stressors, such as neighbour competition, pathogen infection and herbivore attack [5–7]. This may result in allelochemical responses to neighbouring plants, reduced plant susceptibility to pathogen attack or the prevention of grazing by herbivores [8, 9]. However, relatively few studies have sought to understand the mechanisms and effects of stress-induced root-secreted metabolites on rhizosphere microbiota [10, 11].

Among root-secreted metabolites, stress-derived hormones are a class of small bioactive molecules. In addition to regulating plant physiological and morphological responses [12, 13], a growing number of studies have demonstrated that these phytohormones also sculpt the root microbiome [14, 15]. For instance, the secretion of root salicylic acid (SA) is involved in plant neighbour detection and shapes the root microbiome by modulating taxonomic groups of bacteria [9, 10]. Similar functions have been reported for jasmonic acid (JA) and its derivatives [16]. In addition to JA and SA, ethylene (ET), a volatile signal, can easily diffuse through gas- and water-filled pores in the soil [17]. It has been demonstrated that ET can influence nodulation in legume-rhizobia symbioses and arbuscular mycorrhizal root colonization [18–22]. These findings suggest that ET is a chemical cue used to monitor and interact with soil-specific microbial species near growing roots. Gaseous compounds have a wide effective range in soil for the long-distance attraction of bacteria to roots [23]. However, which rhizobacteria are affected by ET and how ET controls the rhizosphere microbial community assembly remains unknown.

When two species co-exist, heterospecific metabolites are important cues for neighbour detection and subsequently trigger complex plant response strategies [5, 9]. Cyanide commonly occurs in over 3000 plant species, including important crop plants, such as maize, wheat and cassava [24, 25]. Exposure to cyanide can shorten plant embryo dormancy and induce ET production in seedlings [26, 27]. In modern intensive agro-ecosystems, cyanide-containing crops are intercropped with legumes (intercropping: a farming practice involving two or more crop species or genotypes growing together and coexisting for a period of time [28, 29]), but very few studies have focused on the chemical linkage between cyanide-containing plants and legumes [28–30]. Whether cyanide from cyanide-rich plants act as a chemical cue to influence ET signalling in non-cyanide-containing plants remains to be uncovered.

To explore whether the belowground chemical interactions between interspecific plants triggers legume rhizosphere microbial re-assembly, we grew cyanide-containing cassava plants together with the legume peanut, which are commonly co-cultivated in subtropical areas [31]. We hypothesized that (1) ET emission from peanut roots can be triggered by the production of cyanide by neighbouring cassava; (2) similar to SA and JA, ET, a volatile hormone, can attract specific microbial species and reshape the microbiota of the plant rhizosphere; (3) the reconstructed microbial community may improve peanut fitness. To evaluate these hypotheses, a 3-year field experiment of peanut and cassava intercropping and peanut monocropping systems and corresponding pot and hydroponic experiments were conducted. We find that neighbour cyanide can be a chemical cue to induce peanut root ET emissions. Using 16S rRNA high-throughput sequencing analysis from the field and ethylene application cultures, we demonstrate that ET modulates the abundance of an actinobacterial *Catenulispora* species, which functioned as a keystone of the re-assembled microbial network in the intercropped peanut rhizosphere. The reshaped microbial community increased the accumulation of available nutrients in the peanut rhizosphere. Our results reveal a novel chemical dialogue between a focal plant’s roots and its microbiota, entering a partnership that improved the focal plant’s fitness when grown with heterospecific plant neighbours.

## Results and discussion

### Peanut traits and rhizosphere nutrient characteristics when co-cultured with cassava in the field

**Fig. 1:**
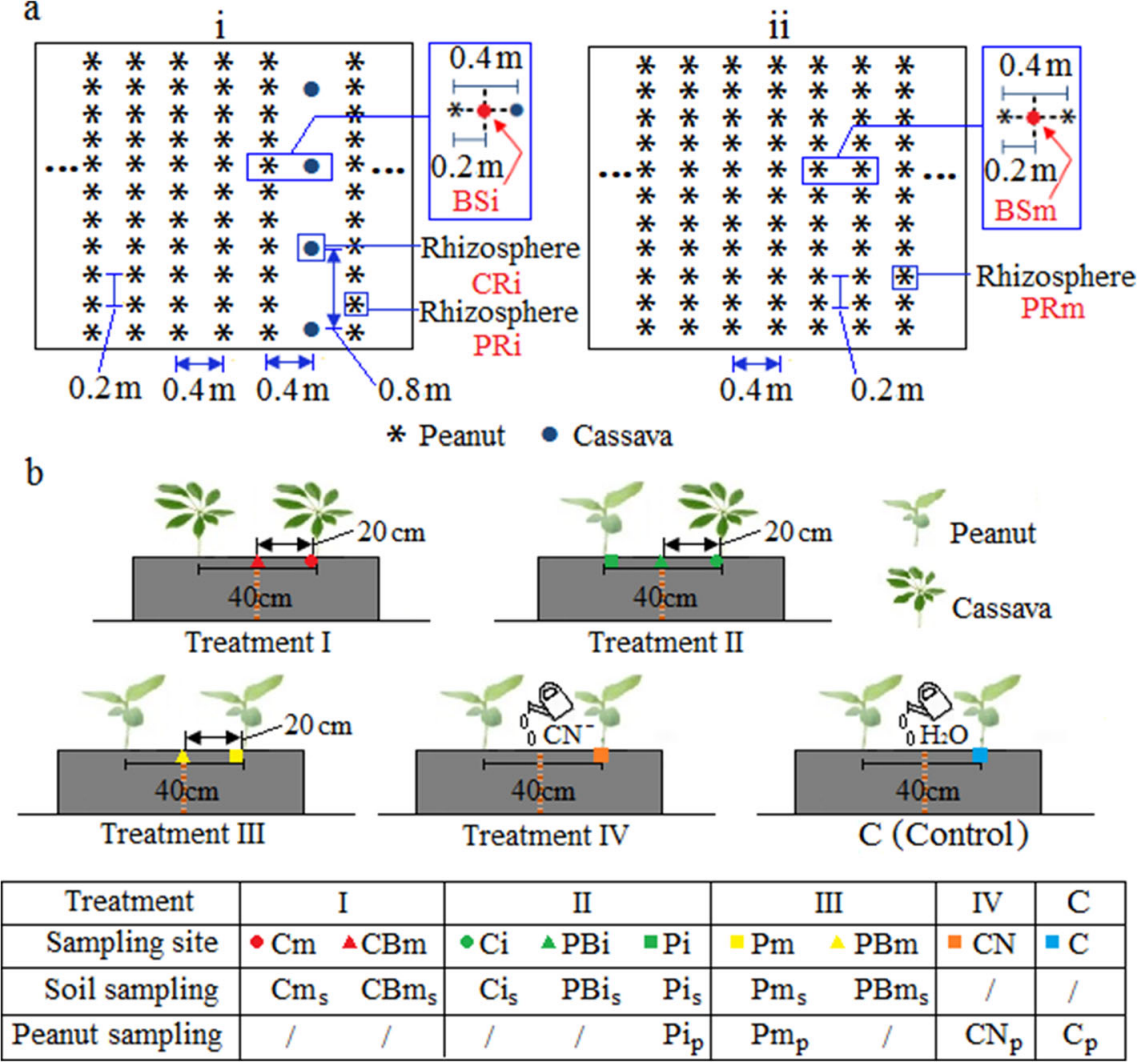
Diagram of intercropping and monocropping sampling sites in the field and pot experiments. **a** The peanut and cassava intercropping system (i), and the peanut monocropping system (ii) in the field. The intercropping combination included a 2.0-m peanut strip (five rows of peanut, with a 0.4 m interrow distance) and a 0.4-m cassava strip (one row of cassava). The interplant distance within the same column was 0.2 m. In the monocropping system, the interrow distance was 0.4 m, and the interplant distance within the same column was 0.2 m. CRi, BSi and PRi represent soils from the cassava rhizosphere, the bulk soil and the peanut rhizosphere in the intercropping system; PRm and BSm represent soils from the peanut rhizosphere and the corresponding bulk soil in the monocropping system. **b** Schematic of the intercropping and monocropping pot experiments. Pairs of plants were grown together in a pot separated into compartments with a wire mesh screen filter (red dotted line). Treatment I, cassava monocropping; treatment II, peanut and cassava intercropping, treatment III, peanut monocropping; treatment IV, peanuts were cultured with the addition of cyanide dilution; and control, peanuts were cultured with the addition of water. The rhizosphere and bulk soil collected from the corresponding coloured sites are indicated by the site name plus lowercase “s”; the peanut plants collected from the sites are indicated by the site name plus lowercase “*p*”

To cope with the biotic stresses from heterospecific species, focal plants display a myriad of plastic responses to optimize fitness [32, 33]. Here, the yield (seed production) of peanut in the peanut-cassava intercropping system (Fig. 1a) was similar to that in the monocropping system through an increase in the number of aboveground branches (*P* = 0.004), the number of pods per plant and the full fruit rate (*P* < 0.001) (Table 1). However, to gain these advantages, peanut reduced its biomass (*P* < 0.001), specifically the aboveground biomass (plant height, *P* < 0.001; and the ratio of aboveground biomass to belowground biomass, *P* < 0.001) (Table 1). In intercropped peanut individuals, seed production determines the reproductive capacity of the offspring [33, 34]. When peanut was co-cultured with neighbouring cassava, it invested more in seeds than in aboveground tissues. Such physiological alteration in the focal plant community parallels the co-existence strategy for plants with a short stature in Evolutionary Game Theory [35, 36].

**Table 1:** Peanut growth and production yield in the two cropping systems

Index	Monocropping system	Intercropping system	*F*	*P*
Plant height (cm)	46.67 ± 3.51	40.66 ± 2.16	33.95	< 0.001
Chlorophyll content	59.56 ± 0.55	60.17 ± 1.36	2.41	0.131
Number of aboveground branches	9.72 ± 2.34	16.16 ± 4.14	15.67	0.004
Biomass per plant (g)	12.96 ± 0.87	11.19 ± 0.96	29.88	< 0.001
Belowground biomass per plant (g)	2.35 ± 0.35	1.76 ± 0.25	29.45	< 0.001
A/B	4.60 ± 0.52	5.51 ± 0.77	15.68	< 0.001
Number of pods per plant	15.87 ± 3.90	21.20 ± 2.35	41.76	< 0.001
Full fruit rate (%)	70.38 ± 6.12	86.21 ± 5.43	58.36	< 0.001
Peanut yield (kg ha^−1^)	282.57 ± 34.28	304.75 ± 30.15	3.78	0.061

Values are the means (*n* = 16) ± the standard deviation of the mean. *A/B* ratio of aboveground biomass to belowground biomass. *F* and *P* were used to show the significant difference based on one-way ANOVA

In addition to plant physiological changes, significant changes of nutrients in the rhizosphere also attracted our attention (Table S1 in Additional file 1). The rhizosphere is the main micro-domain from which plants acquire resources [37]. The overyielding of peanut individuals did not cause a decline of nutrient supply in the rhizosphere. In contrast, intercropped peanut rhizospheres showed improved soil physiochemical properties, including soil organic carbon (SOC), total nitrogen (TN), ammonia nitrogen (NH_4_^+^-N), nitrate nitrogen (NO_3_^−^-N), total phosphorus (TP) and available phosphorus (AP), compared with monocropped peanut rhizospheres (*P* < 0.05) (Table S1 in Additional file 1). Since soil microbes play central roles in rhizosphere nutrient release and conversion [37], the differences in nutrient supply between inter- and monocropping peanut rhizospheres may imply an alteration of the microbial communities in the two systems.

### Neighbouring cyanide triggers peanut root ethylene production

**Fig. 2:**
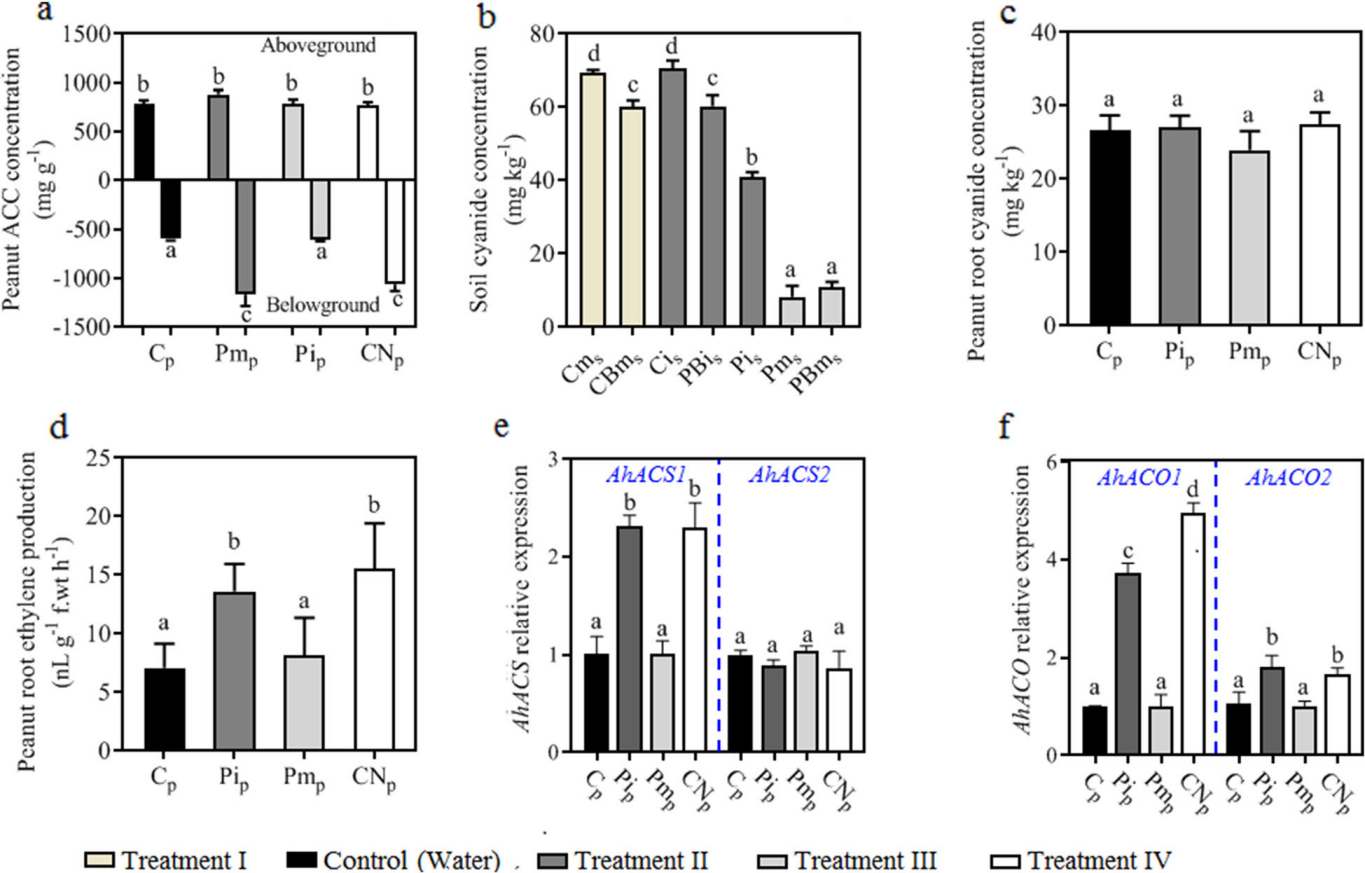
Effects of soil cyanide on root ethylene synthesis and release. **a** The 1-aminocyclopropane-1-carboxylic acid (ACC, ethylene precursor) concentration in aboveground and belowground peanut tissues in different treatments. **b** Cyanide concentration in cassava and peanut rhizosphere soils and corresponding bulk soils. **c** Cyanide concentration in peanut roots. **d** Ethylene (ET) production in peanut roots in different treatments. **e** qRT-PCR detection of ACS gene (*AhACS1* and *AhACS2*) expression in peanut roots in different treatments. **f** qRT-PCR detection of ACO gene (*AhACO1* and *AhACO2*) expression in peanut root in different treatments. Treatment I (cassava monocropping), Cm_s_ and CBm_s_ represent cassava rhizosphere and bulk soil, respectively. Treatment II (cassava and peanut intercropping), Ci_s_, PBi_s_ and Pi_s_ represent soil from the cassava rhizosphere, the bulk soil and peanut rhizosphere; Pi_p_ represents peanut plants from treatment II. Treatment III (peanut monocropping), Pm_s_ and PBm_s_ represent soil from the peanut rhizosphere and bulk; Pm_p_ represents peanuts from treatment III. Treatment IV (peanut monocropping with exogenous CN^−^ addition), CN_p_ represents peanuts from treatment IV. Control (peanut with water addition), C_p_ represents peanuts from control. Data of soil and plant cyanide are mean values + SD for triplicate; data of plant ACC concentration, ET production and gene expression are values + SD for 9 replicates. Error bars with different letters indicate a significant difference according to one-way analysis of variance (ANOVA) with Tukey’s HSD test (*P* < 0.05)

In the intercropping system, peanut individuals allocated more resources to seed production. This physiological response is always coordinated by phytohormonal signalling [38, 39]. By monitoring phytohormone levels in peanut belowground and aboveground tissues (Fig. 1b), we found that peanut if intercropped with cassava (Pi_p_) reduced the belowground zeatin and indole-3-acetic acid (IAA) concentrations and aboveground jasmonic acid (JA) concentrations while stimulating aboveground gibberellin (GA) and root 1-aminocyclopropane-1-carboxylic acid (ACC) production (*P* < 0.05) (Fig. S1 in Additional file 1, Fig. 2a). Similar responses could be mimicked by exogenous cyanide addition (CN_p_ in treatment IV) (Fig. S1 in Additional file 1, Fig. 2a). Unlike other food crops, the levels of cyanide production in cassava is depended on plant cultivars and age, with an average of 577 mg kg^−1^ f.wt in the outer peel (2–5 mm) of the roots [40]. Studies have demonstrated that cyanide can rapidly cross membranes and acts as a regulator to induce ET by modifying ACC synthase and oxidase [26, 41]. However, it has never been reported to participate in natural plant-plant chemical recognition. Here, we found that the soil cyanide concentration formed a steep gradient from the cassava rhizosphere (Ci_s_) to the peanut rhizosphere (Pi_s_) when the two species were co-cultured, and the cyanide content in the peanut rhizosphere (Pi_s_) was still four times higher than that in the peanut rhizosphere of the monocultured system (Pm_s_ and PBm_s,_ *P* < 0.05) (Fig. 2b). However, cyanide concentration in peanut tissue was not affected (Fig. 2c). Along with soil cyanide concentration, root ACC in intercropped peanut (Pi_p_) was two times that of the control (C_p_) and monocropped peanut (Pm_p_). Exogenous cyanide addition proved to increase ACC concentration of peanut root (CN_p_) to a level similar to that of intercropped peanut (Pi_p_) (Fig 2a). Cyanide from neighbouring cassava positively affected the belowground ACC production by peanut. 1-Aminocyclopropane-1-carboxylic acid (ACC) is the direct precursor of ethylene (ET) in plants [42, 43]. Higher ACC in peanut roots corresponded to higher ET production (Fig. 2d).

Acera et al. [44] reported that some soil microorganisms have the potential to transform cyanide to amino acids (such as β-cyanoalanine), which may be further metabolized to ET [13]. To eliminate the interference of soil microorganisms on ET production, quantitative real-time PCR (qRT-PCR) was used to track gene expression related to 1-aminocyclopropane-1-carboxylic acid synthase (ACS) and 1-aminocyclopropane-1-carboxlic acid oxidase (ACO). The expression of the *AhACS1*, *AhACO1* and *AhACO2* transcripts, which probably encode peanut ACS and ACO proteins, was significantly higher in the intercropped and cyanide-treated peanut roots (Pi_p_ and CN_p_) (Fig. 2e, f). Similar differences were found between the cyanide addition treatments and the control in the hydroponic experiments (Fig. S2 in Additional file 1). When plant species co-exist, heterospecific compounds become chemical cues for focal plants to recognize competitors [45]. Here, cyanide from neighbouring cassava was shown to activate the peanut ET production in response to the neighbour belowground.

As the central hormone regulator, ethylene (ET) triggers a range of physiological adaptations [46–48]. Higher ET causes plants to invest more in reproduction under harsh conditions [13]. The presence of a heterospecific neighbour maximized peanut seed production at the expense of plant biomass (Table 1). This optimized performance is the objective in agriculture but suppressed naturally [35, 36].

### Ethylene chemically regulates rhizosphere microbial diversity and composition

**Fig. 3:**
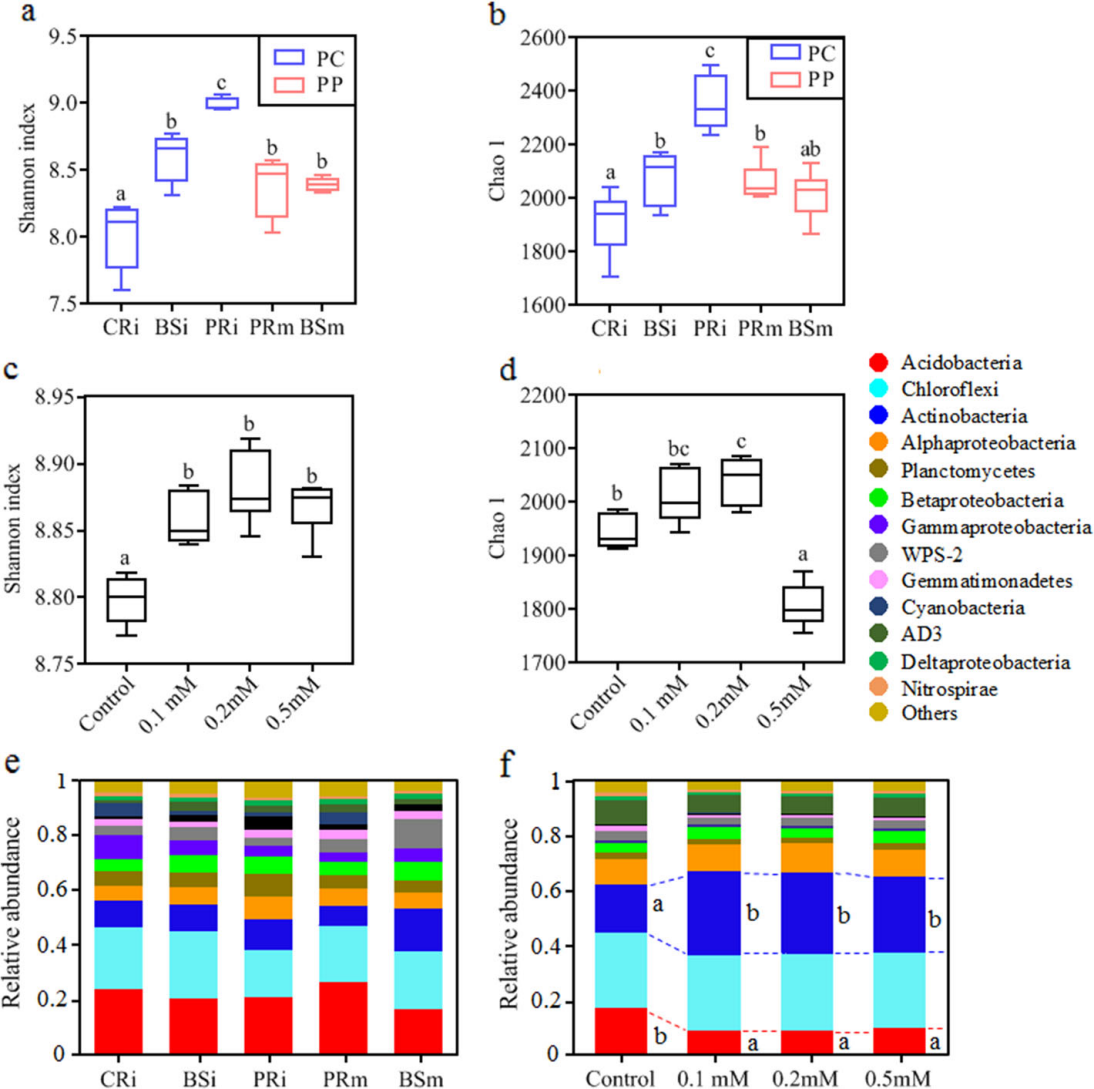
α-Diversities of soil microbiota in the field and ethylene (ET) addition incubation system. **a**, **b** Shannon and Chao 1 indices of the microbiota of rhizosphere soil from cassava and peanut and the corresponding bulk soils in the inter- (blue columns) and monocropping (pink columns) field systems. **c**, **d** Shannon and Chao 1 indices of the microbiota in different ET concentration treatments. **e**, **f** Phylum-level distribution of microbial composition in the field and in the ET addition treatments. Error bars with different letters indicate a significant difference according to one-way analysis of variance (ANOVA) with Tukey’s HSD test (*P* < 0.05)

Conventional agricultural wisdom might suggest that the increased peanut yield was associated with high nutrient consumption from the soil [49, 50]; however, in contrast to our expectations, available nutrients, including ammonia nitrogen (NH_4_^+^-N) and available phosphorus (AP), were higher in the intercropped peanut rhizosphere than in the monocropped peanut rhizosphere (Table S1 in Additional file 1). Microorganisms are the decomposers to mineralize organic matter in soil, and their activity, diversity and composition determine the amount of available nutrients to plants [37]. Using 16S rRNA high-throughput sequencing, we found that the lowest microbial Shannon and Chao 1 indices were observed in the cassava rhizosphere (CRi) (*P* < 0.05) (Fig. 3a, b), which may be attributed to the large amounts of cyanide accumulating around cassava roots (Fig. 2b). A high concentration of cyanide has been reported to be toxic to microbes [25, 40]. In contrast, the peanut rhizosphere in the intercropping system (PRi) showed the highest microbial diversity (Shannon and Chao 1 indices, *P* < 0.05) (Fig. 3a, b). This finding differs from conventional views, because rhizosphere microbial communities are generally less diverse than those in bulk soil [51, 52]. In the intercropping systems, however, root microbiota often show a higher diversity [53, 54]. Depending on microbial substrate preferences, secondary metabolites of plants shape the rhizosphere microbial community [11]. Here, neighbouring cassava increased ethylene production in the belowground portion of peanut. In order to find out if increasing ethylene (ET) concentration directly altered rhizobacterial α-diversity, we applied exogenous ethylene to rhizosphere soil of monocropped peanut in an incubation experiment. We found that compared with an untreated control, ethylene treatment, at the 0.1–0.2 mM concentration level, significantly increased rhizosphere bacterial α-diversity (Shannon and Chao 1 indices, Fig. 3c, d).

Overall, the phyla Acidobacteria, Chloroflexi, Actinobacteria, Alphaproteobacteria, Planctomycetes, Betaproteobacteria and Gammaproteobacteria were present at high relative abundances (average relative abundance > 5%) in the field (Fig. 3e). Interestingly, only the abundance of Actinobacteria and Acidobacteria differed significantly between peanut rhizospheres in intercropping (PRi) and monocropping (PRm) systems (*P* < 0.001). The same effect could be mimicked by exogenous ethylene addition: as actinobacterial abundance increased, acidobacterial abundance decreased with ethylene addition compared to the control (*P* < 0.05) (Fig. 3f). This result is in line with Lebeis [10], who showed that the phytohormone salicylic acid (SA) affects the composition of the root microbiome of *Arabidopsis thaliana*, leading to reduced representation of Acidobacteria and enrichment of Actinobacteria. Here, we paid special attention to the influence of ethylene on the composition of the rhizosphere microbial communities. Similar to SA, ethylene has the potential to alter microbial community assembly of plant roots, which subsequently may feedback on plant phenotypic traits [13]. Such a mechanism could be exploited to improve sustainable plant production systems.

**Fig. 4:**
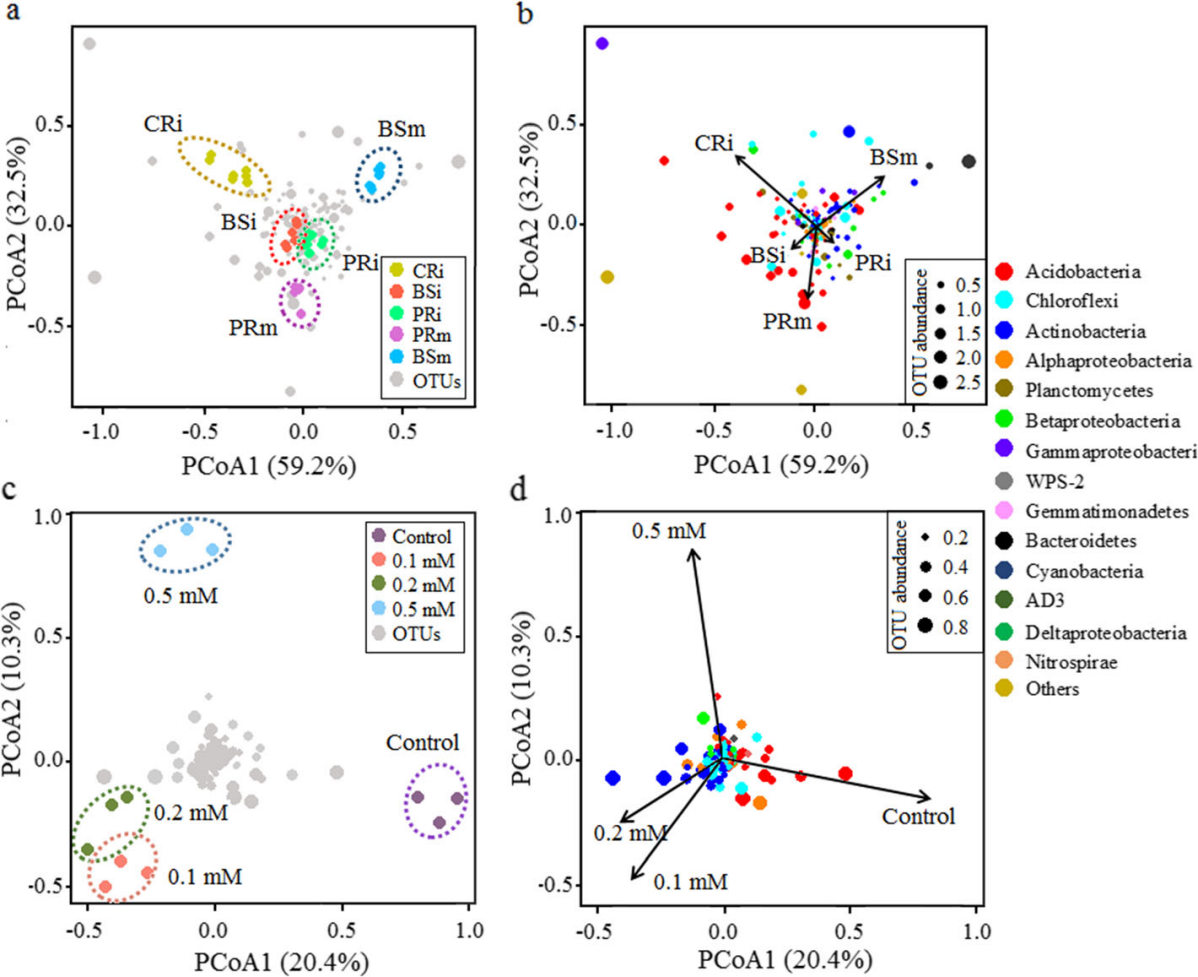
Principal coordinate analysis (PCoA) of the weighted UniFrac values among the field and ethylene addition incubation samples based on Bray-Curtis distances. **a** Bacterial community composition in the field by PCoA. The microbiota is clearly clustered into four groups that are marked with four coloured dotted ellipses. **b** The dominant OTU (relative abundance > 0.1%) scores of the bacterial community in the field according to the PCoA. **c** Bacterial community composition in the ET addition treatments by PCoA. **d** The dominant OTU (relative abundance > 0.1%) scores of the bacterial community in the ET addition treatments according to the PCoA. The arrows indicate the centroid of the constrained factor. Circle sizes represent the relative abundances of bacterial OTUs, and colours were assigned to different phyla. CRi, cassava rhizosphere soil in the interspecific experiment; BSi, bulk soil 20 cm away from the peanut and cassava plants; PRi, peanut rhizosphere soil in the interspecific experiment; PRm, peanut rhizosphere soil in the intraspecific experiment; BSm, bulk soil 20 cm away from peanut plants

To further elucidate differences in community turnover between the soil and rhizosphere microbiota in the field, we analysed β-diversity using Bray-Curtis distances in principal coordinate analysis (PCoA) based on weighted UniFrac metrics (Fig. 4). In the peanut monocropping system, the rhizosphere (PRm) and bulk soil (BSm) were separated (*P*_ANOSIM_ < 0.01) (Fig. 4a), indicating a clear plant rhizosphere effect on the bacterial community assembly [4, 28, 37]. However, in the presence of neighbouring cassava, the cyanide-influenced bulk soil-associated and peanut rhizosphere bacteria (BSi and PRi) appeared to converge, but were still well separated from those of the monocultures (PRm and BSm) and cassava rhizosphere (CRi) (*P*_ANOSIM_ < 0.01) (Fig. 4a). Similar patterns of clustering by planting system were confirmed by hierarchical clustering (Fig. S3 in Additional file 1). Differences in community composition between PRi and PRm were mainly due to the reduction of Acidobacteria (Fig. 4b). This was consistent with the results of the ethylene addition experiment, where concentrations of 0.1 mM and 0.2 mM ET led to a replacement of Acidobacteria by Actinobacteria clustered away from the control along the first coordinate axis (*P*_ANOSIM_ < 0.01) (Fig. 4c, d). Fu [55] confirmed that many species of Actinobacteria have the potential to utilize ethylene (ET) as a source for their carbon metabolism. This may explain the increasing relative abundance of Actinobacteria in intercropped and ethylene-treated monocropped peanut rhizospheres.

### Ethylene regulates the rhizosphere network through effects on specific microbial taxa

Microorganisms do not exist in isolation but interact with others in nature by positive, negative or neutral ecological interactions [56]. Microbial co-occurrence and network analysis provide a promising approach to investigate these various types of interactions in microbial communities [56, 57]. In this study, we constructed co-occurrence networks using random matrix theory (RMT) to determine the differences in bacterial assemblages in rhizospheres of the different plant species and bulk soil [58, 59]. All values of the calculated modularity index were larger than 0.4 (Table 2), suggesting typical module structures [60]. Overall, crop planting showed a marked effect on the soil microbial network: average path distance (GD), the average clustering coefficient (*avg*CC) and modularity of the empirical networks were higher than those of corresponding, identically sized random networks (Table 2). Plant rhizosphere network connectivity and complexity were first well described as properties of rhizosphere bacterial assemblages by Shi et al. [58]. Here, we found that plant rhizosphere assemblages (in CRi, PRi and PRm) formed more connected and more complex networks with fewer nodes but more connections (edges) between nodes compared with the bulk soil. Accordingly, the densities of the connections (graph density) in the rhizosphere increased in the plant rhizosphere compared with the corresponding bulk soil. In the networks, the ratio of positive to negative connections (edges) was 1.2 in PRi and 2 in PRm (Table 2), indicating that more competitive or inhibitive connections in the intercropped peanut rhizosphere.

**Table 2:** Topological properties of the empirical molecular ecological networks of microbial communities in treatments

Network metrics	Treatments				
	CRi	BSi	PRi	PRm	BSm
Empirical networks					
Number of nodes	121	148	139	121	146
Number of edges	309	309	357	289	283
Number of positive correlations	184	180	194	194	147
Number of negative correlations	125	129	162	95	136
Ratio of positive to negative correlations	1.47	1.40	1.20	2.04	1.08
Average connectivity (*avg*K)	5.11	4.18	5.14	4.78	3.88
Average path distance (GD)	4.50	4.66	4.13	5.06	5.17
Average clustering coefficient (*avg*CC)	0.30	0.27	0.31	0.33	0.26
Graph density	0.043	0.028	0.037	0.040	0.027
Number of modules^a^	5	6	8	6	9
Modularity	0.48	0.64	0.53	0.67	0.60
Random networks					
GD ± SD	3.05 ± 0.07	3.59 ± 0.06	3.12 ± 0.05	3.22 ± 0.06	3.62 ± 0.07
*avg*CC ± SD	0.10 ± 0.02	0.03 ± 0.01	0.07 ± 0.01	0.06 ± 0.01	0.05 ± 0.01
Modularity±SD	0.36 ± 0.01	0.36 ± 0.01	0.37 ± 0.01	0.31 ± 0.01	0.28 ± 0.01

^a^The number of modules with ≥ 5 nodes in the networks. *CRi* cassava rhizosphere soil in the intercropping experiment, *BSi* bulk soil 20 cm away from the peanut and cassava plants, *PRi* peanut rhizosphere soil in the intercropping experiment, *PRm* peanut rhizosphere soil in the monocropping experiment, *BSm* bulk soil 20 cm away from peanut plants in monocropping system

**Fig. 5:**
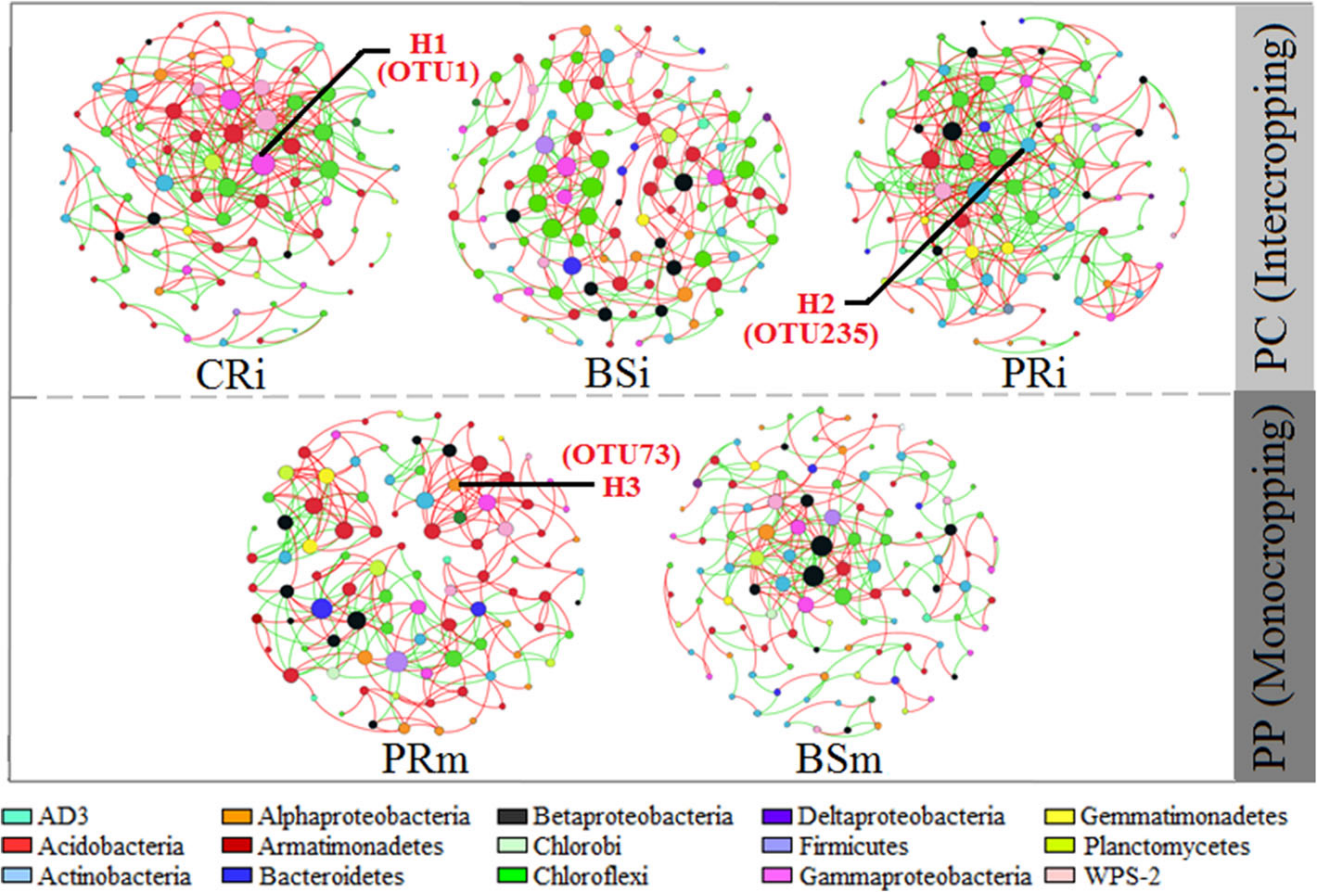
Plant rhizosphere and the corresponding bulk soil networks in the inter- and monocropping systems. Networks represent random matrix theory co-occurrence models derived from 8 biological replicates at each site, where nodes represent OTUs, and the edges between the nodes indicate significant correlations. A green edge indicates negative covariation between two individual nodes, while a red edge indicates positive covariation. The colours of the nodes indicate major phyla (subphyla for Proteobacteria). In each panel, the size of each node is proportional to the number of connections (i.e. node degree). Nodes marked “H1, H2 and H3” represent the identified module hubs as follows: H1, OTU1 *Dokdonella*; H2, OTU235 *Catenulispora*; and H3, OTU73 *Pseudolabrys*

Microbial communities can harbour keystone taxa, whose removal can cause a dramatic shift in microbiome structure and functioning [57, 60]. Keystone taxa in network analysis can be computationally identified as hubs with a high within-module degree *Zi* (*Zi ≥* 6.2 indicates that the nodes are “well-connected” to other nodes in the module) [59, 61–63]. In this study, bacterial module hubs affiliated with *Dokdonella* (OTU1, H1 belonging to Gammaproteobacteria), *Catenulispora* (OTU235, H2 belonging to Actinobacteria) and *Pseudolabrys* (OTU73, H3, belonging to Alphaproteobacteria) were respectively identified in the cassava, intercropped peanut and monocropped peanut rhizosphere samples (CRi, PRi and PRm) (Table 3, Fig. S4 in Additional file 1). No hub was found in the bulk soil (BSi and BSm) (Fig. 5). In the peanut rhizosphere, *Catenulispora* sp. replaced *Pseudolabrys* sp. as a keystone taxon in intercropping and showed more negative correlations to other taxa in the network (Table 3, Fig. 5). *Catenulispora* belongs to Actinobacteria, which is a phylum that serves as a source of highly diverse antibiotics [64, 65]. *Catenulispora* is known to produce the antibiotics cacibiocin A and B, which are inhibitors of bacterial type II topoisomerases, such as DNA gyrase and DNA topoisomerase IV in Acidobacteria [66]. To a certain extent, this can explain the decrease in the acidobacterial abundance in the rhizosphere of the peanut intercropping system (Fig. 3e, f, Fig. 4b, d).

**Table 3:** Nodes identified as hubs in the networks in the inter- and monocropping systems

Networks	ID	Role	Abundance (%)	Degree	Negative edges	Phylum	Genus	*Z* value^a^	*P* value^a^	Cluster coefficient
CRi	OTU1	Hub	6.935	22	15	Gammaproteobacteria	*Dokdonella*	2.601	0.430	0.416
PRi	OTU235	Hub	0.183	15	12	Actinobacteria	*Catenulispora*	2.582	0.124	0.410
PRm	OTU73	Hub	0.209	7	0	Alphaproteobacteria	*Pseudolabrys*	2.516	0.198	0.377

^a^The topological role of each node is determined according to two properties: *Z*_*i*_ the within-module connectivity, and *P*_*i*_ the among-module connectivity

**Fig. 6:**
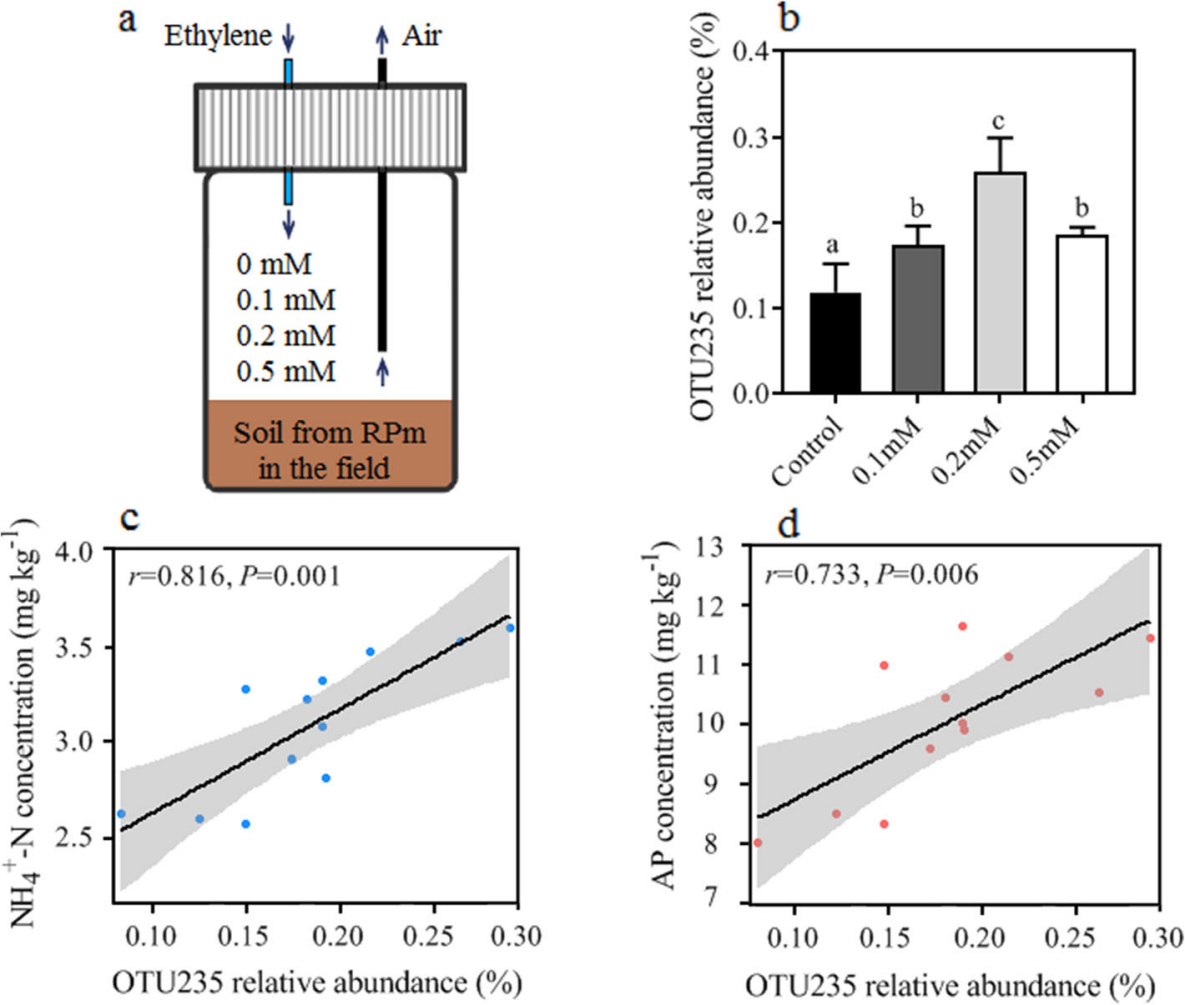
The variance of the relative abundance of the keystone and the correlation between its abundance and soil available nutrient content in ethylene addition treatments. **a** Layout of the exogenous ethylene addition test. Thirty grams of soil from the PRi treatment were placed in the bottom of a sterilized bottle. Then, 200 mL of 0.1 mM, 0.2 mM and 0.5 mM standard ethylene gas (ethylene and air mixed) were injected into the glass bottle (*V* = 100 mL) to fully replace the air. For the control (0 mM), the injection gas was replaced with air. After injection, the tubes were immediately sealed and incubated at 26 °C for 7 days for microbial community detection. **b** Dynamics of relative abundance of OTU235 (module hub H2 in RPi network) in different ethylene level treatments. Columns with different colours indicate significant differences (*P* < 0.05) according to one-way analysis of variance (ANOVA) followed by Tukey's HSD test (*P* < 0.05). **c**, **d** Significant relationship between OTU235 abundance and available nutrients (NH_4_^+^-N and AP) in the ethylene addition treatments (*P* < 0.01)

We further used indicator species analysis to identify operational taxonomic units (OTUs) specifically associated with ethylene addition and found that actinobacterial indicators appeared in the 0.1 mM and 0.2 mM ethylene addition treatments (Table S2 in Additional file 1). Indicator species analysis is a method determined by Dufrene-Legendre [67] to identify species preference for environmental changes. In the control (no ethylene addition) and in high ethylene addition (0.5 mM) treatments, no actinobacterial indicator was found (Table S2 in Additional file 1). Interestingly, among actinobacterial indicators, OTU235 (*Catenulispora* sp.), which was the keystone in the network of the intercropped peanut rhizosphere, again turned out as indicator taxon in the 0.2 mM ethylene (ET) treatment (Table S2 in Additional file 1). Its relative abundance was significantly dependent on ethylene concentration (Fig. 6a, b). To date, there is no evidence of direct interactions between *Catenulispora* sp. and plant phytohormones, but species belonging to Catenulisporaceae have been reported to act as plant-associated bacteria strengthening root colonization by producing the enzyme ACC deaminase, which controls the endogenous ethylene (ET) levels [68, 69]. In addition to the increase in *Catenulispora* sp. relative abundance, the soil ammonium nitrogen (NH_4_^+^-N) and available phosphorus (AP) concentrations increased (*r* = 0.816 and *r* = 0.733, *P* < 0.01) (Fig. 6c, d). This may be attributed to the positive effect of the re-assembled microbial community on soil phosphorus (index of acid phosphatase activity, *P* < 0.05) and organic nitrogen (indices of urease activity and L-glutamate activity, *P* < 0.05) mineralization (Fig. S5 in Additional file 1).

### Influence of soil properties, plant traits and the rhizosphere microbial community on peanut production

**Fig. 7:**
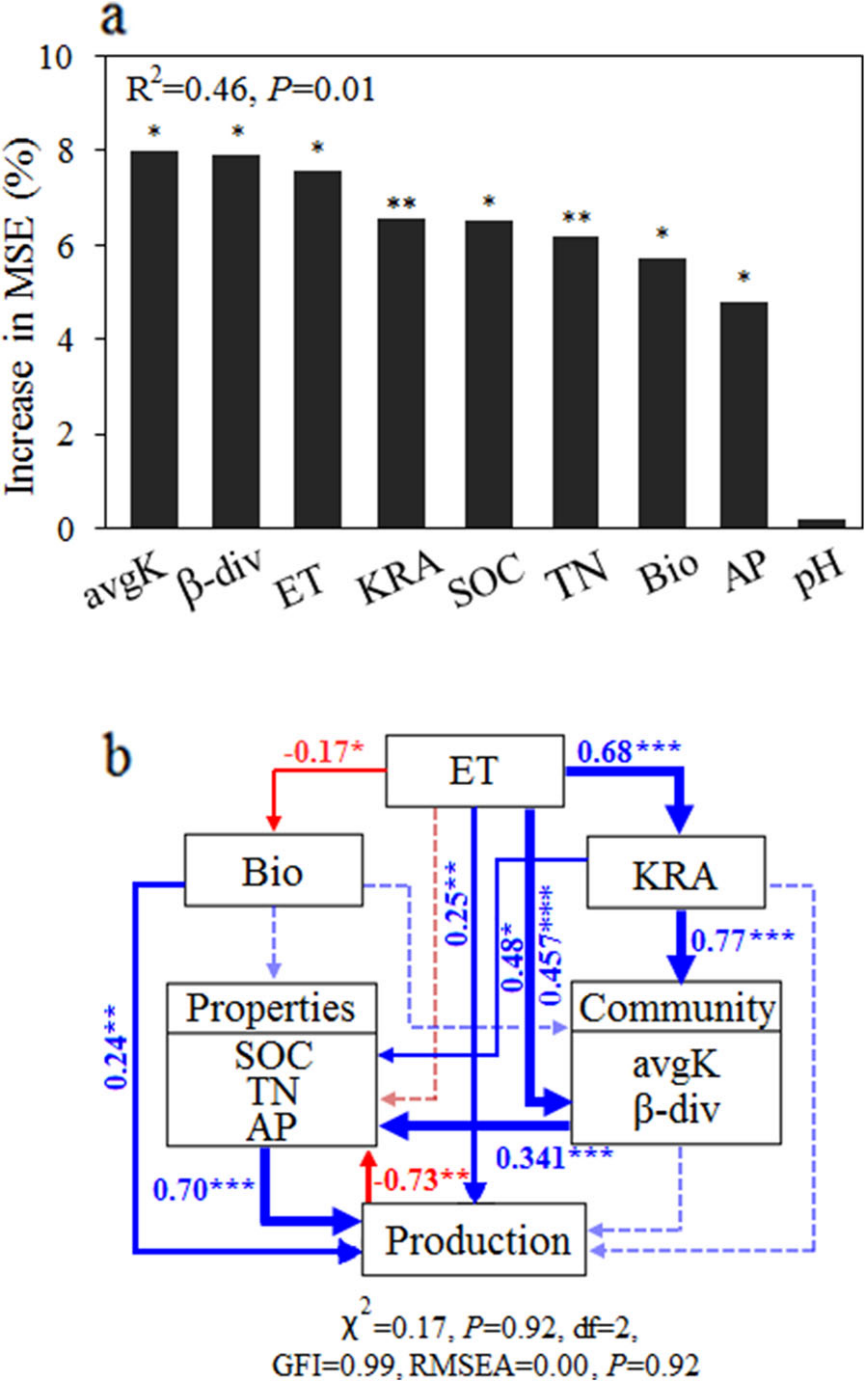
Direct and indirect effects of ethylene on peanut seed production. **a** Mean predictor importance of factors related to plant fitness based on random forest analysis. **b** Direct and indirect effects of ethylene emission on seed production using structural equation modelling (SEM). The significance levels of each predictor in the random forest analysis are as follows: *P < 0.05 and **P < 0.01. The blue and red arrows in the structural equation model indicate positive and negative relationships, respectively, and dotted arrows represent nonsignificant paths (*P* > 0.05). Numbers adjacent to arrows are standardized path coefficients, and the path width indicates the strength of significant standardized path coefficients. The first principal coordinate (PCoA1, which explained 59.3% of the variation) is used to represent the composition of the bacterial β-diversity. ET, ethylene emission; Bio, plant biomass; KRA, keystone relative abundance; avgK, average network connectivity; β-div, bacterial β-diversity; SOC, soil organic carbon; TN, soil total nitrogen; AP, soil available phosphorus

To investigate the potentially important predictors of peanut seed production, we conducted random forest modelling with the soil properties (including TN, SOC, AP and pH), plant traits (including ethylene emission and plant biomass), and bacterial community (including β-diversity, relative abundance of keystone taxa and average network connectivity) [70]. The model indicated that the most important predictor of seed production was average network connectivity, followed by bacterial β-diversity, ethylene (ET) emission, relative abundance of keystone taxa, SOC, TN, plant biomass and AP (*P* < 0.05) (Fig. 7a). Soil pH had no influence on plant seed production (*P* = 0.33) (Fig. 7a). We then used structural equation modelling (SEM) to identify the potential direct and indirect effects of soil properties, plant traits and the microbial community on seed production (Fig. 7b). Structural equation modelling (SEM) is a powerful statistical modelling technique that is widely used in the behavioural sciences and ecological research [71]. It can be viewed as a combination of factor analysis and regression or path analysis. In this study, soil properties, ethylene (ET) production and peanut biomass were positively related to peanut seed production (*P* < 0.01). Although ethylene slightly reduced plant biomass (*P* < 0.05), it modulated the microbial community structure by affecting keystone species abundance (*P* < 0.01). The resulting microbial re-assembly showed a direct positive correlation with the soil properties (*P* < 0.001), suggesting that microbial community reconstruction did benefit nutrient mineralization (Fig. 7b).

**Fig. 8:**
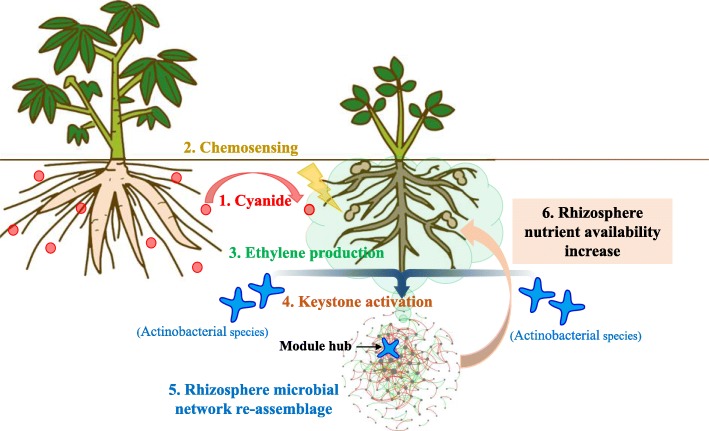
The overview of the mechanism by which peanut mediates rhizosphere microbiota to increase available nutrients in the cassava-peanut intercropping system. Cyanide exposure belowground induces ethylene production in peanut roots, and ethylene mediates actinobacterial species to reshape rhizosphere microbiota, which promote peanut seed production by increasing available nutrient content

Keystone species serve as gatekeepers in the ecological functions of the bacterial community [59, 60]. When detecting cyanide from a neighbour, root ethylene from the peanut directly affects the abundance of the gatekeeper, which leads to a dramatic shift in the community composition of the peanut rhizosphere microbiota, and likely further increases seed production by enhancing the accumulation of available nutrients in the rhizosphere (Fig. 8). During the long-term co-evolution of plants and microorganisms, plants have developed mechanisms to process signals, thus triggering an optimized response to maximize resistance at minimal costs [13]. Signal integration between plants and microbes is a result of killing two birds with one stone, stimulating plant physiological responses and causing plants and microbes to form a coherent unit to shift plant responses towards or away from adaptation to specific situations [15]. In the past, these views were mostly speculative [13, 15]. Here, using a series of experiments, we demonstrated that ethylene not only participates in the recognition of heterospecific plant neighbours, but also helps plants to regulate rhizosphere microbial communities to improve plant fitness in the process of plant interspecific interactions.

## Conclusions

Cyanide produced by neighbouring plants is a chemical cue that increases root ethylene emission from the legume peanut. This phytohormone signal led to an increased relative abundance of specific actinobacterial species, thereby reshaping the whole rhizosphere microbial network. This led to an improved availability of essential nutrients and a shift in plant allocation towards increased seed production at the expense of plant biomass. Our results reveal a novel function of a stress-derived plant signal to re-shape microbial community assembly in the rhizosphere and establishing strategic plant-microbe partnership enhancing plant fitness in intercropping agro-ecosystems.

## Materials and methods

### Field experiment site

The field site is located at the Red Soil Ecological Experimental Station of the Chinese Academy of Sciences in Yingtan, Jiangxi Province, China (28° 15′ N, 116° 5′ E). The site is located at an altitude of 79 m a.s.l., with a mean annual temperature of 17.6 °C and a mean annual precipitation of 1795 mm. The total solar radiation is 6514.2 MJ m^−2^ year^−1^, and the potential evaporation is 1318 mm. The frost-free period is 262 days. The soil type is classified as acid loamy clay derived from Quaternary red clay (Udic Ferralsols in the Chinese Soil Taxonomy and Ferric Acrisols in the FAO classification system) with a pH (water) of 4.9. In the top 25 cm of the soil profile, the soil organic carbon (SOC) content was 10.23 g kg^−1^, the total nitrogen (TN) content was 0.90 g kg^−1^, the available phosphorus (AP) content was 34.15 mg kg^−1^, and the available potassium (AK) content was 235.11 mg kg^−1^.

### Inter- and monocropping field experimental design

The experiment was conducted from 2013 to 2016 and included the following two treatments: (i) PC, a peanut (*Arachis hypogaea*, a legume) and cyanide-containing cassava (*Manihot esculenta* Crantz) intercropping system, and (ii) PP, a peanut monocropping system (Fig. 1a). The design of the PC treatment (i) included a 2.0-m peanut strip (five rows of peanut, with a 0.4 m interrow distance) and a 0.4 m cassava strip (one row of cassava). The interplant distance within the same row was 0.2 m for peanut and 0.8 m for cassava. In the PP treatment (ii), the interrow and interplant distances were 0.4 m and 0.2 m, respectively, which made the peanut density identical to that in a comparable area in the PC treatment (Fig. 1a). Each treatment was replicated four times. All plots received 120 kg ha^−1^ nitrogen fertilizer (urea containing 46% N), 90 kg ha^−1^ phosphorus (calcium superphosphate containing 12.5% P_2_O_5_) and 135 kg ha^−1^ K_2_O (potassium chloride containing 60.0% K_2_O). Each individual plot was 10 × 8 m (length × width), and a ridge (with a width of 0.4 m and a height of 0.3 m) separated adjacent plots. Cassava stems (20 cm in length) were transplanted on 15–18 April and harvested on 10–15 November, while the peanuts were sown on 15–20 April and harvested on 15–20 August. All plots were irrigated and weeded during the growing period. The yields of peanut and cassava were determined at harvest in 2016.

### Soil and plant sampling in the field

Field soil samples were collected as follows: (1) PC intercropping: peanut rhizosphere soil (PRi), cassava rhizosphere soil (CRi), and bulk soil (BSi) 20 cm away from the peanut and cassava roots; (2) PP monocropping: peanut rhizosphere soil (PRm) and bulk soil (BSm) 20 cm away from peanut roots (Fig. 1a). Soil that tightly adhered to the plant roots was defined as rhizosphere soil [63]. Due to the small amount of rhizosphere soil per plant, we randomly selected six plant roots that were pooled into a single rhizosphere soil sample. In total, 40 soil samples (5 sites × 2 replicates for each plot × 4 plot replicates) were immediately sieved (4 mm) in the laboratory. Any visible living plant material was manually removed. Five grams of each soil sample was stored at − 80 °C for microbial molecular analysis, and the rest was stored at 4 °C for chemical analyses. Standard methods were used to characterize soil chemical properties (see the details in Methods S1 of Additional file 1). An additional sample of 500 kg of soil was collected at a 5–20-cm depth within a 1.5 × 2 m area in the PP treatment after the peanuts were harvested and stored at 20 °C for the soil pot experiment.

Sixteen peanut plants in each treatment (4 biological replicates × 4 plot replicates) were randomly selected to determine the chlorophyll content in situ (from a single leaf at the top of the plant) using SPAD 502 plus (Konica, Tokyo, Japan) and to measure plant height and aboveground branching. Plant biomass was determined after drying at 65 °C to a constant weight. All the soil and plant samples were collected on 29 May 2016 at the peanut flowering stage.

### Pot experiment to detect peanut ethylene production

To identify the metabolites involved in interspecific interactions, inter- and monocropping pot treatments (treatments I–III) were performed as shown in Fig. 1b. Each pot was 80 × 40 × 15 cm (length × width × depth). A wire mesh screen (0.05 mm) was placed in the centre of a pot to create two compartments (40 × 40 × 15 cm in length × width × depth). In treatment I, two cassava individuals were planted in the two compartments in a single pot; in treatment II, cassava and peanut individuals were planted in the two compartments; and in treatment III, two peanut individuals were planted in the two compartments (Fig. 1b). Based on the variation in the soil cyanide concentration and peanut phytohormones in the inter- and monocropping pot treatments, an additional exogenous cyanide (CN^−^) treatment (treatment IV) was performed to identify the hormonal response of peanut to soil cyanide concentrations. For treatment IV, 50 mL of 4 mg L^−1^ CN^−^ in water (diluted cyanide standard) was sprayed on the soil surface before 24 h of destructive plant sampling. For the control (C), the solution was replaced with distilled water. Each treatment comprised nine biological replicates.

Plants were germinated separately and transplanted to the pots when their aboveground height reached 10 cm. They were grown at a temperature of 25–26 °C, 65–70% relative humidity, and a 12/12 h light/dark photoperiod with a light intensity of 10,000 lx. Each pot contained 16 kg (dry soil weight) of soil from the peanut monocropping system in the field (PP). After 20 days of transplantation, plants were destructively sampled.

For soil cyanide measurement, 10 g of each soil sample from seven sites (Fig. 1b) in treatments I–III (treatment I: Cm_s_, cassava rhizosphere soil, and CBm_s_, the bulk soil 20 cm away from the cassava roots; treatment II: Ci_s_, cassava rhizosphere soil; Pi_s_, peanut rhizosphere soil; and PBi_s_, the bulk soil 20 cm away from the cassava and peanut roots; and treatment III: Pm_s_, peanut rhizosphere soil, and PBm_s_, the bulk soil 20 cm away from the peanut roots) were collected and determined based on the methods of the U.S. EPA [72]. To detect plant cyanide, 5 g of fresh plant tissue was ground and measured based on the methods of the U.S. EPA [72]. Among nine replicates of each treatment, three replicates were randomly chosen (7 sampling sites × 3 replicates) to collect soil from specific sites.

For peanut phytohormone detection (see the details in Methods S1 of Additional file 1), a total of 36 plant individuals collected from four treatments (treatment II: Pi_p_, intercropped peanut; treatment III: Pm_p_, monocropped peanut; treatment IV: CN_p_, 24 h of cyanide stressed peanut; and control: C_p_, peanut in the control) were immediately divided into below- and aboveground tissues and then frozen in liquid nitrogen for phytohormone determination, including zeatin, gibberellin (GA3), abscisic acid (ABA), auxin (IAA), salicylic acid (SA), jasmonic acid (JA) and ethylene precursor 1-aminocyclopropane-1-carboxylic acid (ACC). Each treatment comprised nine biological replicates, including 36 peanut plants (from 4 treatments × 9 replicates) and 21 soil samples (7 sampling sites × 3 replicates).

### Measurement of ethylene production from peanut roots

Due to strong variations of root 1-aminocyclopropane-1-carboxylic acid (ACC), we examined ethylene (ET) production and its synthesis by gene expression in peanut roots. We repeated treatments II, III, and IV and the control (C) mentioned in the “Pot experiment to detect peanut ethylene production” section. After culturing for 20 days, a total of 36 peanut roots (4 treatments × 9 replicates) were cut and placed in 15 mL glass vials containing 1 mL 0.6% water agar and rapidly sealed with a gas-proof septum according to Wu [73]. After 4 h of incubation in darkness at 30 °C, 1 mL of the gas was withdrawn from the airspace of each vial using a gas-tight syringe (Focus GC, Thermo, Massachusetts, USA) and injected into a gas chromatograph (Focus GC, Thermo) equipped with a capillary column (CP-CarboPLOT P7, California, USA) and flame-ionization detector for ET determination. The production of ethylene (ET) was calculated on the basis of the fresh weight (f.wt) of the root samples [74]. Moreover, 36 other peanut roots (4 treatments × 9 replicates) were collected and frozen immediately in liquid nitrogen for RNA extraction and ACS/ACO gene expression analysis.

### Expression analysis of ACS and ACO transcripts in peanut roots

1-Aminocyclopropane-1-carboxlic acid synthase (ACS) and (1-aminocyclopropane-1-carboxlic acid oxidase (ACO) are key enzymes controlling plant ethylene synthesis. These genes are organ-specific and are differentially regulated by different environmental signals [75]. To date, these genes in peanut have not been reported. Fortunately, based on the ACS/ACO protein sequences in *Arabidopsis thaliana* (TSA contigs GDKN01000001 - GDKN01102303 in NCBI http://www.ncbi.nlm.nih.gov/), we mined three hypothetical and expressed *AhACS*/*AhACO* transcripts in allotetraploid peanut transcript data [75]. Total root RNA was extracted with E.Z.N.A. Total RNA Kit I (OMEGA, GA, USA) and reverse transcribed into cDNA with cDNA Synthesis Kit (Thermo Fisher Scientific, MA, USA) following the manufacturer’s instructions. Quantitative real-time PCR (qRT-PCR) was performed in a 20 μL reaction volume using a CFX connect Real-Time System (Thermo Fisher Scientific, MA, USA) and TB Green^TM^ Premix Ex Taq^TM^ (Takara, Kusatsu, Japan). The peanut Actin gene (Aradu. W2Y55) was used as the internal control, and the relative quantitation of target gene expression among the different experimental conditions was calculated using the comparative Ct method. All the qRT-PCR primers are listed in Table S3 of Additional file 1. All the mRNA data were expressed as percent of the corresponding Actin transcript levels.

### Effect of exogenous cyanide on peanut ethylene production in a hydroponic experiment

To eliminate the interference of soil microbiota with rhizosphere ethylene production, a hydroponic culture was set up (Fig. S2a of Additional file 1). Seeds were surface-sterilized with 0.1% HgCl_2_ for 5 min and washed twice with sterilized-distilled water and then germinated on sterilized water-moistened gauze. Seedlings were cultivated with 1/4 Hoagland’s nutrient solution until the aboveground height reached 10 cm. Plant growth conditions were consistent with those in the pot experiment “Pot experiment to detect peanut ethylene production”. Then, the seedlings were transferred to 4 mg L^−1^ cyanide-containing solutions (cyanide) and 4 mg L^−1^ cyanide + 4 mg L^−1^ CoCl_2_ (cobalt chloride, an inhibitor of ethylene biosynthesis) solutions (cyanide + CoCl_2_). For the control (H_2_O), solutions were replaced with distilled water. Twenty-four and 48 h after incubation, all roots were cut for the measurement of ethylene production and ACS/ACO transcript expression. Each culture was grown in eight replicates for each time point sampling. After incubation for 24 h and 48 h, four replicates were used to detect root ethylene production, and the other four replicates were used for gene expression.

### Soil incubation with the addition of exogenous ethylene

To further determine whether rhizosphere ethylene had the potential to mediate soil-specific microbiota, we incubated peanut rhizosphere soil with different concentrations of ethylene. Thirty grams of soil from the field monocropping system (PP) was placed in a sterilized glass bottle (*V* = 100 mL). Three concentrations, (A) 0.1 mM, (B) 0.2 mM and (C) 0.5 mM ethylene standard, were injected into the bottle to replace the air (Fig. 6a). A control (0 mM) with air injection was established in an identical manner. After injection, the bottles were immediately sealed and incubated in triplicate at 26 °C for 7 days (Fig. 6a). After 7 days, 1 g soil samples were collected for 16S high-throughput sequencing analysis. The rest of the soil was used for the measurements of chemical properties and enzymatic activities (see the details in Methods S1 of Additional file 1). Each treatment was incubated in triplicates.

### Soil DNA extraction and 16S rRNA high-throughput sequencing

Soil samples (1.0 g) from field and incubation experiments were extracted using the FastDNA SPIN Kit (MP Biomedical, California, USA) according to the manufacturer’s instructions. The quantity and purity of DNA were examined with a Nanodrop ND-1000 spectrophotometer (NanoDrop Technologies, Delaware, USA). The V4-V5 region of the bacterial 16S rRNA gene was amplified using the primers 515F and 907R [76]. Each sample was amplified in a 20-μl reaction system, which contained 0.5 μM forward and reverse primers, 1 × Premix Taq DNA polymerase (Takara, Kusatsu, Japan) and 20 ng DNA templates. After an initial denaturation at 95 °C for 3 min, the targeted region was amplified by 20 cycles of 94 °C for 30 s, 55 °C for 30 s, and 72 °C for 30 s, followed by a final extension at 72 °C for 1 min in a thermal cycler (GeneAmp PCR system 2700; Applied Biosystems, New York, USA). Amplicon sequencing libraries were constructed using the MiSeq Reagent Kit v3 according to the manufacturer’s instructions. High-throughput paired-end sequencing was performed on the Illumina MiSeq PE250 platform.

The raw data were screened and trimmed by the QIIME pipeline (version 1.9.0) [77]. To minimize the effects of random sequencing errors, low-quality and ambiguous reads (Phred quality score *Q* < 25 or sequence shorter than 150 bp) were eliminated. Chimeras were filtered with the UCHIME algorithm in the USEARCH package [78, 79]. High-quality sequences were clustered into operational taxonomic units (OTUs) using UCLUST with a similarity threshold of 97% [78]. The sequences were then phylogenetically assigned to taxonomic classifications using the RDP (Ribosomal Data Project database) classifier and were allocated to different levels [80]. Singletons were removed, and all samples were rarefied to 20,000 sequences per sample for further analysis. The sequencing data were deposited in the European Nucleotide Archive of EMBL under the accession number PRJEB22658.

### Soil microbial network construction and keystone identification

Microbial networks were constructed for rhizosphere and bulk soil communities based on OTU relative abundance in the field experiment. Covariations were determined across eight biological replicates to create each network. Random matrix theory (RMT) was used to construct co-occurrence networks by calculating all pairwise Spearman’s rank correlations (*P* < 0.01). Random networks were generated based on the Maslov and Sneppen model [81]. Global network properties were characterized according to Deng et al. [59]. All network analyses were performed using the Molecular Ecological Network Analyses pipeline (http://ieg2.ou.edu/MENA/) written in Java and Perl scripts [59, 82, 83]. To reduce network complexity, we only considered bacterial OTUs with an average abundance > 0.1%. The OTUs detected in more than 75% of the samples were retained in the network. Various indices, including the average clustering coefficient (avg*CC*), average geodesic distance (GD), and size and modularity of the network, were calculated to describe network topologies. Average connectivity (avg*K*) was calculated to measure the complexity of the network structure [59]. The topological role of each node was determined based on two properties: the within-module connectivity (*Zi*) and the among-module connectivity (*Pi*) [61]. All species were sorted into four subcategories on the basis of these simple criteria: peripherals (nodes in the modules with few outside connections, *Zi* < 2.5 and *Pi* < 0.62), connectors (nodes that connect modules, *Pi* > 0.62), module hubs (highly connected nodes within modules, *Zi* > 2.5) and network hubs (highly connected nodes within the entire network, *Zi* > 2.5 and *Pi* > 0.62) [59, 63, 82, 84]. Correlation networks were visualized using Gephi software [85].

### Structural equation modelling

Random forest modelling was conducted to quantitatively assess the important predictors of plant productivity [70, 86], including soil properties (total nitrogen, TN; soil organic carbon, SOC; available phosphorus, AP and pH), plant physiology (ethylene production, ET; peanut biomass, Bio), and the bacterial community (bacterial β-diversity, β-div; keystone relative abundance, KRA; average connectivity, *avg*K). The importance of each factor was evaluated by the decrease in prediction accuracy (that is, an increase in the mean square error (MSE) between observations and predictions) when the data were randomly permuted [86]. This accuracy importance measure was computed for each tree and averaged over the forest (500 trees). These analyses were conducted using the randomForest package [86], and the significance of the model and predictor importance were determined using the rfUtilities and rfPermute packages in the R software, respectively [87, 88].

We then used structural equation modelling to evaluate the direct and indirect relationships between root ethylene and soil properties and plant physiology and bacterial community. We use the *χ*^2^ test (*χ*^2^; the model has a good fit when *χ*^2^ ≤ 2) and *P* values (traditionally ≥ 0.05), goodness-of-fit index and root mean square error of approximation (RMSEA, ≤ 0.05) and *P* values (traditionally ≥ 0.05) to evaluate the structural equation model fit [89]. The best-fitting and most parsimonious model was obtained after excluding all non-significant parameters. All SEM analyses were conducted using the Amos 17.0 software package (Smallwaters, IL, USA).

### Statistical and network analyses

The differences in the peanut traits and the abundance of bacterial phyla in mono- and intercropping systems (two groups) in the field were analysed by one-way ANOVA. Significant differences (*P* < 0.05) in the soil chemical properties in the field and ethylene addition experiments, soil cyanide and plant hormone concentrations, the relative abundances of soil specific phyla, and ACS/ACO transcript expression between treatments in the pot or hydroponic experiments were evaluated by Tukey’s honest significant difference (HSD) test with SPSS 18.0 (SPSS, Chicago, IL, USA).

In this study, we used Shannon and Chao 1 indices to characterize bacterial ɑ-diversity [90], and the data were subjected to ANOVA using Tukey's HSD test at *P* < 0.05. To assess the influence of the different experimental factors on β-diversity, we calculated Bray-Curtis distances and then performed a canonical analysis of principal coordinates (CAP) constrained by the factor of interest and conditioned by the remaining variables [63, 91]. We employed the “capscale” and “permutest” permutation-based testing functions for the CAP analysis and the calculation of the significance values, respectively [63]. Permutational multivariate analysis of variance (PERMANOVA) was conducted to separate and quantitatively evaluate the effects of driving factors on the composition of the soil bacterial community using the “Anosims” function [92, 93]. Bootstrapped trees were constructed using the weighted pair group method with arithmetic mean (UPGMA) based on 1000 hierarchical clusters in the QIIME package [77]. Indicator species that were specifically associated with the different sampling sites were determined using the “labdsv” package in R (version 3.2.1) [67, 94].

## Supplementary information

**Additional file 1: Methods S1.**Soil chemical property and enzymatic activity determination, and peanut phytohormone measurements. **Fig. S1.** Results of the pot experiments conducted for the detection of peanut phytohormone production. **Fig. S2.** Hydroponic culturing to detect the regulation of root ethylene production in peanut by exogenous cyanide. **Fig. S3.** Hierarchical clustering of soil bacterial community in cassava and peanut intercropping (PC) and peanut monocropping (PP) systems based on pairwise Bray-Curtis distances. **Fig. S4.** *Zi-Pi* plot showing the distribution of OTUs based on their topological roles in the intercropping and monocropping networks. **Fig.S5.** Soil enzymatic activity of organic phosphorus and nitrogen mineralization. **Table S1.** Soil chemical properties in the rhizosphere and bulk soil in the intercropping and monocropping systems. **Table S2.** Information regarding the indicators in ethylene incubating samples. **Table S3.** Gene-specific primers used for qRT-PCR.

## Data Availability

The sequences of 16S rRNA genes were deposited in the European Nucleotide Archive of EMBL under the accession number PRJEB22658.
