# Supplementary material for: Root ethylene mediates rhizosphere microbial community reconstruction when chemically detecting cyanide produced by neighbouring plants
Source: Microbiome. 2020 Jan 18;8:4. doi: 10.1186/s40168-019-0775-6 (PMC6969408; doi:10.1186/s40168-019-0775-6)
Supplement: Supplementary file 2 — Additional file 1: Methods S1.Soil chemical property and enzymatic activity determination, and peanut phytohormone measurements. Fig. S1. Results of the pot experiments conducted for the detection of peanut phytohormone production. Fig. S2. Hydroponic culturing to detect the regulation of root ethylene production in peanut by exogenous cyanide. Fig. S3. Hierarchical clustering of soil bacterial community in cassava and peanut intercropping (PC) and peanut monocropping (PP) systems based on pairwise Bray-Curtis distances. Fig. S4. Zi-Pi plot showing the distribution of OTUs based on their topological roles in the intercropping and monocropping networks. Fig.S5. Soil enzymatic activity of organic phosphorus and nitrogen mineralization. Table S1. Soil chemical properties in the rhizosphere and bulk soil in the intercropping and monocropping systems. Table S2. Information regarding the indicators in ethylene incubating samples. Table S3. Gene-specific primers used for qRT-PCR. [file 40168_2019_775_MOESM2_ESM.docx]

**Additional File 1 for**

**Root ethylene mediates rhizosphere microbial community reconstruction when chemically detecting interspecific neighbours**

Yan Chen^1^, Michael Bonkowski^2, 3^, Yi Shen^4^, Bryan S.Griffiths^5^, Yuji Jiang^1^, Xiaoyue Wang^1^ and Bo Sun^1*^

^1^State Key Laboratory of Soil and Sustainable Agriculture, Institute of Soil Science, Chinese Academy of Sciences, No.71 East Beijing Road, Nanjing 210008, China;

^2^Terrestrial Ecology, Institute of Zoology, University of Cologne, Zülpicher Str 47b, Cologne 50674, Germany;

³Cluster of Excellence on Plant Sciences (CEPLAS), University of Cologne;

^4^Institute of Industrial Crops, Jiangsu Academy of Agricultural Sciences, No.50 Zhonglin Street, Nanjing 210014, China;

^5^SRUC, Crop and Soil System Research Group, West Mains Road, Edinburgh EH93JG, UK

Author for correspondence: Bo Sun

Tel: +8602586881203; Fax: +8602586881000, Email: [bsun@issas.ac.cn](mailto:bsun@issas.ac.cn)

**This file includes**

**Methods S1:** Soil chemical property and enzymatic activity determination, and peanut phytohormone measurements.

**Fig. S1:** Results of the pot experiments conducted for the detection of peanut phytohormone production.

**Fig. S2:** Hydroponic culturing to detect the regulation of root ethylene production in peanut by exogenous cyanide.

**Fig. S3:** Hierarchical clustering of soil bacterial community in cassava and peanut intercropping (PC) and peanut monocropping (PP) systems based on pairwise Bray-Curtis distances.

**Fig. S4:** *Zi-Pi* plot showing the distribution of OTUs based on their topological roles in the intercropping and monocropping networks.

**Fig.S5:** Soil enzymatic activity of organic phosphorus and nitrogen mineralization.

**Table S1:** Soil chemical properties in the rhizosphere and bulk soil in the intercropping and monocropping systems.

**Table S2:** Information regarding the indicators in ethylene incubating samples.

**Table S3:** Gene-specific primers used for qRT-PCR.

**Methods S1**

**Soil chemical property determination**

Soil chemical variables were measured as follows: pH was determined with a glass electrode and a water-to-soil ratio of 2.5:1 (v:w). The SOC content was determined by the Walkley-Black wet digestion method [1]. The TN and nitrate and ammonium nitrogen (NO_3_^-^-N and NH_4_^+^-N, respectively) were measured by the Kjeldahl method (Bremner, 1965). The total phosphorus (TP) was digested with HF-HClO_4_, and AP was extracted with sodium carbonate and sodium bicarbonate and then determined with the molybdenum blue method [2], and the AK was determined by flame photometry after extraction with ammonium acetate [3].

**Soil enzymatic activity determination**

Soil acid phosphatase (ACP) and alkaline phosphatase (ALP) activity were assayed using p-nitrophenyl (p-NP) phosphate as the substrate with the buffer adjusted to pH 6.5 and 11.0, respectively [4]. After incubation, the absorption was measured at 405 nm, and ACP and ALP activity were expressed as mg p-NP g^−1^ soil h^−1^. Soil urease activity was measured by incubating 5 g soil with 5 ml of 10 % urea solution for 24 h at 37 °C. The formation of ammonium was determined spectrophotometrically at 578 nm and the activity was expressed as NH _4_ ^+^-N μg g^−1^ dry weight soil 24 h^−1^ [5]. L-glutaminase was measured following the H_2_SO_4_ titration method [6].

**Peanut phytohormone measurements**

For the phytohormone measurements, 100 mg of plant material was ground into power in liquid nitrogen and extracted with 1.0 mL pre-chilled methanol:H_2_O:formic acid (7.9:2:0.1, v:v:v) overnight at 4°C. The suspension was centrifuged at 13,000 rpm for 20 min at 4°C, and the solid residue was re-extracted and re-centrifuged and then the two supernatants were pooled. The supernatants were passed through an Oasis MAX strong anion-exchange column (Waters, Massachusetts, USA) to remove interfering lipids and some of the plant pigments and then dried under nitrogen gas. The residue was dissolved in 100 μL methanol.

For the measurement of 1-aminocyclopropane-1-carboxylic acid (ACC), 100 mg of the ground power was mixed with 1 mL 80% methanol (v:v) for 1 h at -20°C. The suspension was centrifuged at 24,000 rpm for 20 min, and 0.9 mL of supernatant was transferred to a clean tube and mixed with 0.9 mL H_2_O. The mixture was subjected to further purification using a DEAE Sephadex A-25 column with 1 mL pre-chilled methanol:H_2_O:formic acid (80:14:6, v:v:v). The eluent was freeze dried and re-dissolved in 100 μL methanol.

The dissolved suspension was subjected to LC-MS/MS with an AB Sciex 5500 QTRAP spectrometer (AB Sciex, Toronto, Canada). The LC-MS/MS was operated in negative mode with electrospray as the ionization source. The separation was performed on a Waters ACQUITY HSS T3 (100 mm × 2.1 mm, 1.8 μm) column. Gradient elution was applied with a mobile phase of methanol and 0.1% aqueous formic acid at a flow rate of 0.3 mL min^-1^. The column temperature was maintained at 40°C, and the injection volume was 5 µL.

The calibration standards included a mixed phytohormone standard solution containing zeatin, gibberellin (GA3), abscisic acid (ABA), auxin (IAA), salicyclic acid (SA) and Jasmonic acid (JA) standards (Sigma-Aldrich, Missouri, USA) at concentrations of 0.1, 1, 5, 10, 20, 40, 60, 80 and 100 ng mL^-1^ for each phytohormone standard in the mixed solution. The 1-aminocyclopropane-1-carboxylic acid (ACC) calibration standard was processed at the same concentration levels from 0.1 to 100 ng mL^-1^. The content of each phytohormone was calculated based on the standard curves in units of ng per mg fresh weight using Analyst software 1.6.

**References**

1. Jackson ML. Soil Chemical Analysis. Englewood Cliffs, NJ: Prentice-Hall; 1956.

2. Olsen SR, Cole C, Watanabe FS, Dean L. Estimation of Available Phosphorus in Soils by Extraction with Sodium Bicarbonate. USA: USDA Press; 1954.

3. Kanehiro Y, Sherman GD. Fusion with sodium carbonate for total elemental analysis. In: Black CA, editor. Methods of Soil Analysis II. USA: American Society of Agronomy; 1965. p. 952–958.

4. Tabatabai MA. Soil enzymes. In: Weaver RW, Angle JS, Bottomley PS, editor. Methods of Soil Analysis, Part 2, Microbiological and Biochemical Properties.USA: Soil Science Society of America; 1994. p. 775–833.

5. Klose S, Tabatabai MA. Urease activity of microbial biomass in soils. Soil Biol Biochem. 1999; 31: 205–211.

6. Frankenberger WT, Tabatabai MA. L-glutaminase activity of soils. Soil Biol Biochem. 1991; 23: 869-874.

**
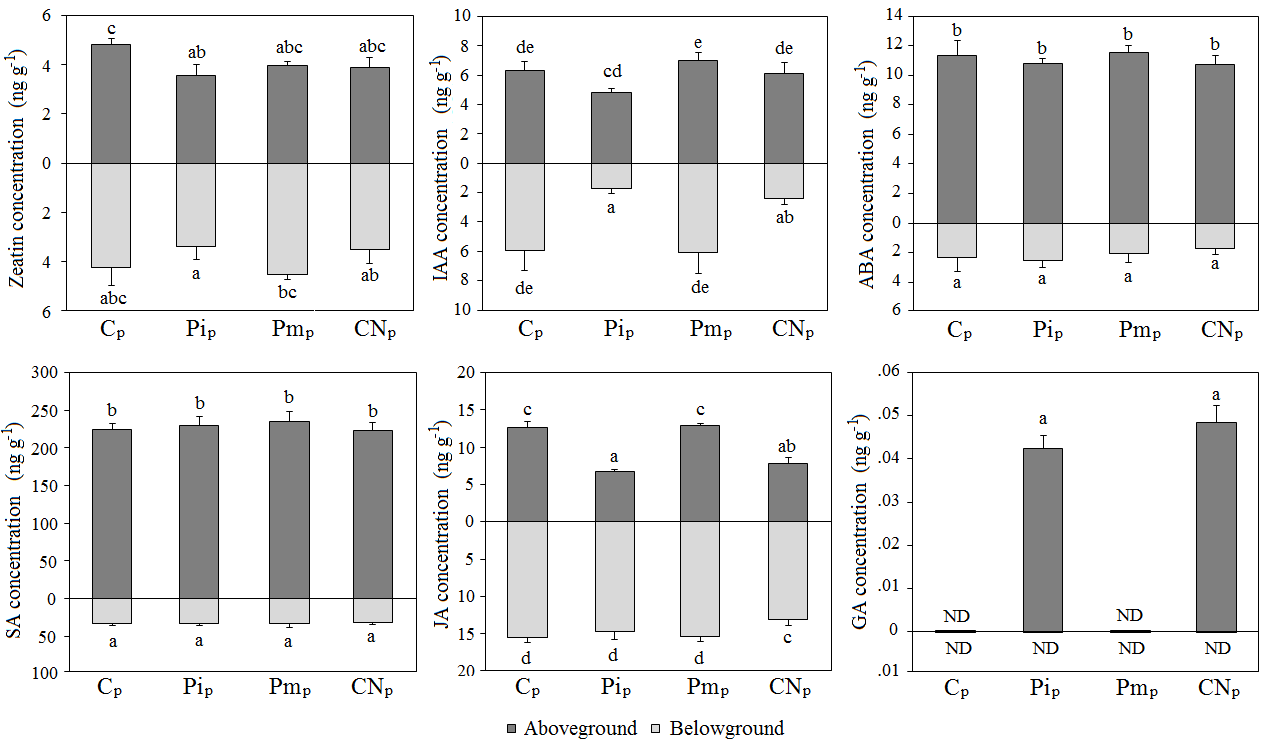
Fig. S1** Results of the pot experiments conducted for the detection of peanut phytohormone production. Pi_p_ and Pm_p_, peanut plants in the inter- and mono-cropping pot experiments (Treatment II and III), respectively; C_p_, peanut plants under water addition (Control); CN_p_, peanut plants collected from exogenous CN^-^ application (Treatment IV). Data are mean values + SD for triplicates (n=3). Error bars with different letters indicate a significant difference according to one-way analysis of variance (ANOVA) followed by Tukey's HSD test (*P* < 0.05). GA, gibberellin; IAA, indole-3-acetic acid; ABA, abscisic acid; SA, salicylic acid; JA, jasmonic acid; ND, not detected.

**
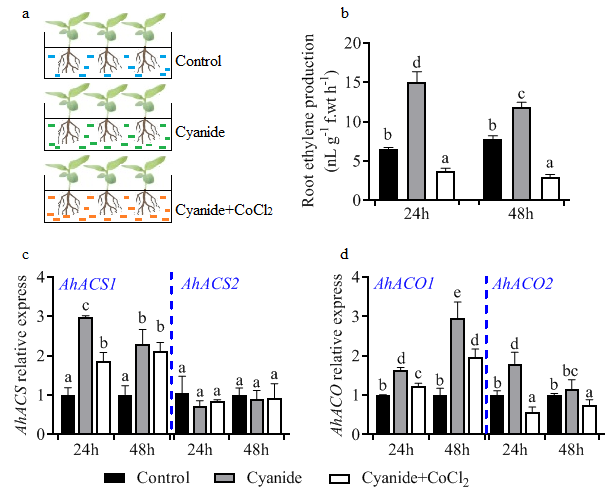
**

**Fig. S2** Hydroponic culturing to detect the regulation of root ethylene production in peanut by exogenous cyanide. **a**, diagram of hydroponic culturing design. **b**, root ethylene production after 24h, and 48h of treatments. **c-d**, qRT-PCR verification of root *AhACS* (c) and *AhACO* (d) genes expressed after 24h, and 48h of incubation. Results are based on three biological replicates (*n*=4); data are mean values + SD. Error bars with different letters indicate a significant difference according to one-way analysis of variance (ANOVA) with Tukey's HSD test (*P* < 0.05).


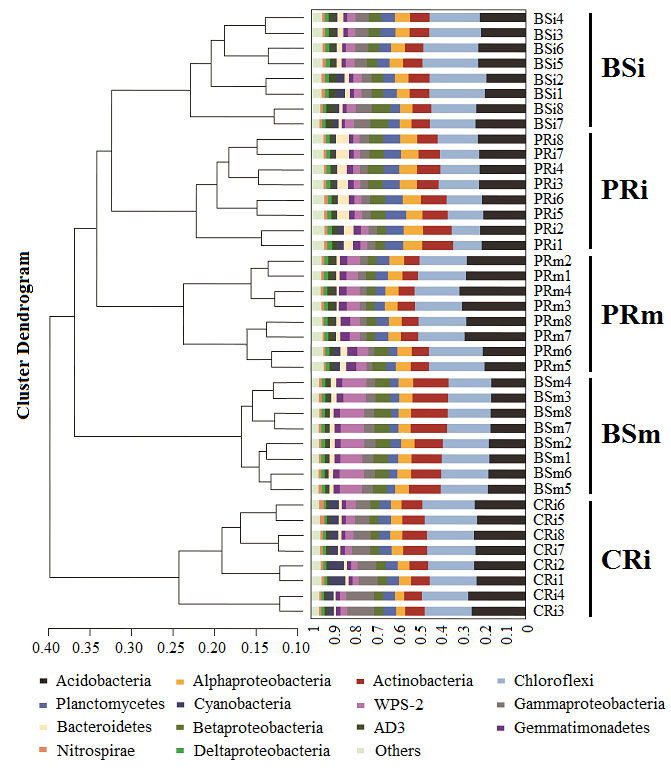


**Fig. S3** Hierarchical clustering of soil bacterial community in cassava and peanut intercropping (PC) and peanut monocropping (PP) systems based on pairwise Bray-Curtis distances. The samples clustered into five groups. CRi, BSi and PRi represent soils from the cassava rhizosphere, the bulk and the peanut rhizosphere in intercropping system; PRm and BSm represent soils from the peanut rhizosphere and the corresponding bulk in monocropping system.


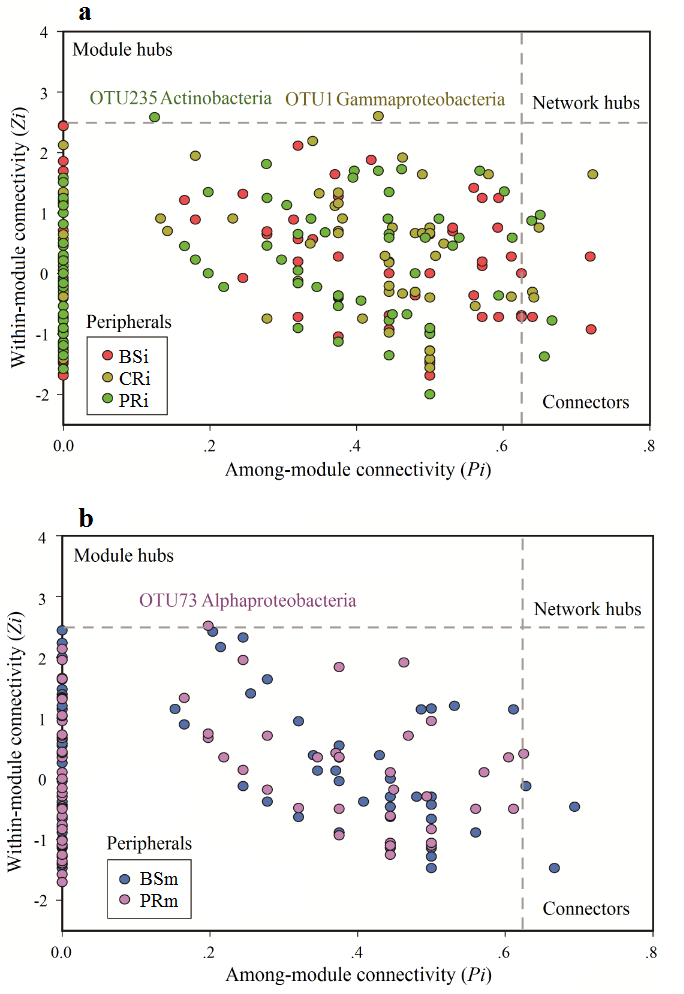


**Fig. S4** *Zi-Pi* plot showing the distribution of OTUs based on their topological roles in the intercropping (**a**) and monocropping (**b**) networks. Each symbol represents an OTU in the bacterial network. Module hubs have *Zi*>2.5, whereas connectors have *Pi*>0.62. The phylogenetic affiliations of the hubs are listed on the plots. Detailed taxonomic information on the module hubs is provided in Table 3.


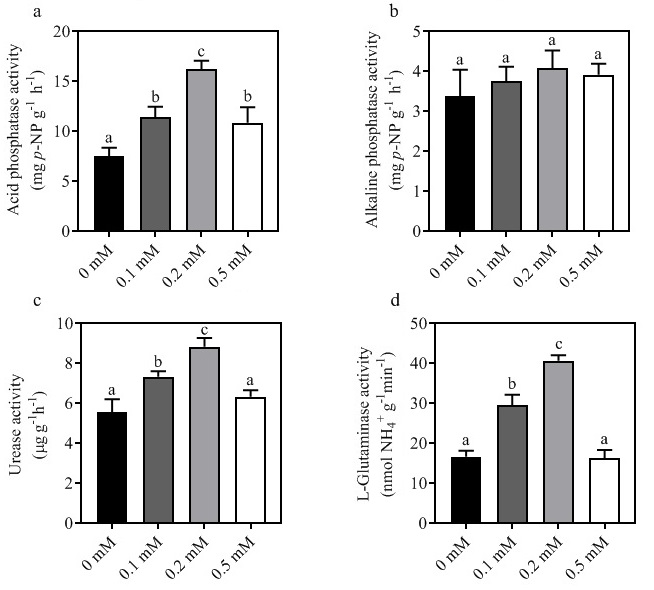


**Fig. S5** Soil enzymatic activity of organic phosphorus and nitrogen mineralization. **a-b**, soil acid and alkaline phosphatase activities in different ethylene addition cultures. **c**-**d**, soil urease, L-glutaminase activities in different ethylene addition cultures. Results are based on three biological replicates; data are mean values + SD. Error bars with different letters indicate a significant difference according to one-way analysis of variance (ANOVA) with Tukey's HSD test (*P* < 0.05).

**Table S1 Soil chemical properties in the rhizosphere and bulk soil in the intercropping and monocropping systems**

| **Treatments** | **SOC**  **(g kg^-1^)** | **TN**  **(g kg^-1^)** | **TP**  **(g kg^-1^)** | **AK**  **(mg kg^-1^)** | **NH_4_^+^-N**  **(mg kg^-1^)** | **NO_3_^-^-N**  **(mg kg^-1^)** | **AP**  **(mg kg^-1^)** | **pH** |
| --- | --- | --- | --- | --- | --- | --- | --- | --- |
| CRi | 10.01±0.57ab | 0.89±0.01b | 0.68±0.02c | 219.63±19.73a | 3.56±0.08d | 9.14±0.20c | 73.32±1.41c | 4.66±0.01a |
| BSi | 9.42±0.36a | 0.95±0.01c | 0.76±0.02d | 221.42±15.55a | 3.07±0.09a | 8.85±0.10c | 66.08±2.36b | 4.79±0.03d |
| PRi | 11.25±0.66c | 1.04±0.01e | 0.78±0.01d | 224.22±14.07a | 3.44±0.08c | 11.50±0.70d | 75.65±1.49d | 4.95±0.01e |
| PRm | 10.06±0.60ab | 0.83±0.01a | 0.58±0.02b | 232.67±9.96ab | 3.32±0.08b | 5.13±0.59b | 35.23±1.59a | 4.77±0.01c |
| BSm | 10.48±0.11bc | 0.99±0.01d | 0.49±0.02a | 243.56±18.74b | 3.64±0.08d | 3.82±0.24a | 33.89±1.92a | 4.75±0.01b |

Values are the means (n=8) ± the standard error of the mean. Values in the same column followed by a lowercase letter indicate a significant difference according to one-way analysis of variance (ANOVA) followed by Tukey's HSD test (P < 0.05). Soil chemical properties include SOC, soil organic carbon; TN, total nitrogen content; TP, total phosphorus content; AK, available potassium; NH_4_^+^-N, ammonium nitrogen; NO_3_^-^-N, nitrate nitrogen; AP, available phosphorus; pH and moisture.

**Table S2 Information regarding the indicators in ethylene incubating samples**

| **OTU ID** | **Abundance (%)** | **Treatment** | **Indicator Value** | **P**  **value** | **Phylum** | **Genus** | **Identity (%)** |
| --- | --- | --- | --- | --- | --- | --- | --- |
| OTU1852 | 0.304 | 0.1mM | 0.3438 | 0.047 | Betaproteobacteria | Glaciimonas | 99 |
| OTU2168 | 0.633 | 0.1mM | 0.3343 | 0.018 | Chloroflexi | Kallotenue | 84 |
| OTU126 | 0.126 | 0.1mM | 0.3091 | 0.043 | Planctomycetes | Gemmata | 89 |
| OTU1389 | 0.331 | 0.1mM | 0.3559 | 0.042 | Betaproteobacteria | Burkholderia | 98 |
| OTU156 | 0.133 | 0.1mM | 0.3229 | 0.038 | Bacteroidetes | Mucilaginibacter | 98 |
| OTU216 | 0.222 | 0.1mM | 0.3571 | 0.027 | Actinobacteria | Tetrasphaera | 99 |
| OTU187 | 1.602 | 0.1mM | 0.3112 | 0.026 | Actinobacteria | Moorella | 83 |
| OTU210 | 0.198 | 0.1mM | 0.3323 | 0.042 | Chloroflexi | Thermaerobacter | 87 |
| OTU121 | 0.259 | 0.1mM | 0.3218 | 0.036 | Chloroflexi | Thermaerobacter | 86 |
| OTU138 | 0.239 | 0.1mM | 0.3282 | 0.018 | Betaproteobacteria | Ramlibacter | 99 |
| OTU233 | 0.248 | 0.1mM | 0.3342 | 0.039 | Actinobacteria | Arthrobacter | 99 |
| OTU3611 | 1.524 | 0.1mM | 0.3449 | 0.023 | Betaproteobacteria | Massilia | 99 |
| OTU399 | 0.479 | 0.1mM | 0.4962 | 0.012 | Cyanobacteria | Diphyscium | 99 |
| OTU235 | 0.174 | 0.2mM | 0.323 | 0.012 | Actinobacteria | Catenulispora | 99 |
| OTU1037 | 0.12 | 0.2mM | 0.3801 | 0.032 | Actinobacteria | Actinoallomurus | 97 |
| OTU1102 | 0.161 | 0.2mM | 0.3494 | 0.016 | Alphaproteobacteria | Blastochloris | 97 |
| OTU179 | 0.098 | 0.2mM | 0.3464 | 0.041 | Chloroflexi | Dictyobacter | 87 |
| OTU40 | 0.472 | 0.2mM | 0.324 | 0.039 | Alphaproteobacteria | Bradyrhizobium | 99 |
| OTU184 | 0.631 | 0.2mM | 0.301 | 0.02 | Actinobacteria | Conexibacter | 96 |
| OTU345 | 0.187 | 0.2mM | 0.4122 | 0.018 | Chloroflexi | Dictyobacter | 88 |
| OTU360 | 0.117 | 0.2mM | 0.4961 | 0.014 | Acidobacteria | Candidatus Koribacter | 95 |
| OTU139 | 0.228 | 0.5mM | 0.3661 | 0.026 | Betaproteobacteria | Ralstonia | 99 |
| OTU612 | 0.124 | 0.5mM | 0.3004 | 0.02 | Chloroflexi | Dictyobacter | 87 |
| OTU733 | 0.115 | 0.5mM | 0.4079 | 0.024 | Acidobacteria | Candidatus Solibacter | 99 |
| OTU263 | 0.248 | 0.5mM | 0.3252 | 0.044 | Chloroflexi | Kallotenue | 85 |
| OTU292 | 0.296 | 0.5mM | 0.3194 | 0.029 | Acidobacteria | Occallatibacter | 85 |
| OTU135 | 0.214 | 0.5mM | 0.388 | 0.019 | Bacteroidetes | Mucilaginibacter | 99 |
| OTU109 | 0.344 | Control | 0.3235 | 0.037 | Firmicutes | Bacillus | 99 |
| OTU30 | 0.141 | Control | 0.413 | 0.026 | Acidobacteria | Holophaga | 88 |
| OTU1174 | 0.096 | Control | 0.3939 | 0.02 | Acidobacteria | Candidatus Koribacter | 92 |
| OTU1597 | 0.637 | Control | 0.3305 | 0.03 | Chloroflexi | Actinomyces | 85 |
| OTU17 | 0.293 | Control | 0.4702 | 0.02 | Acidobacteria | Acidobacterium | 95 |
| OTU45 | 0.069 | Control | 0.3663 | 0.025 | Planctomycetes | Tepidisphaera | 90 |
| OTU2297 | 0.165 | Control | 0.5 | 0.022 | Firmicutes | Tepidanaerobacter | 84 |
| OTU268 | 0.176 | Control | 0.5429 | 0.015 | Firmicutes | Tumebacillus | 99 |
| OTU272 | 0.119 | Control | 0.3386 | 0.042 | Chloroflexi | Dehalogenimonas | 84 |
| OTU298 | 0.1 | Control | 0.3699 | 0.019 | Chloroflexi | Dehalogenimonas | 84 |
| OTU4 | 0.513 | Control | 0.3842 | 0.027 | Firmicutes | Tepidanaerobacter | 84 |
| OTU16 | 0.987 | Control | 0.3777 | 0.017 | Acidobacteria | Acidobacterium | 91 |
| OTU71 | 0.872 | Control | 0.3215 | 0.021 | Firmicutes | Thermosinus | 84 |

OTU that indicator value >0.3 and *P*<0.05 was listed as indicator.

**Table S3 Gene-specific primers used for qRT-PCR.**

| **Gene Name** | **Forward Primer (5'-3')** | **Reverse Primer(5'-3')** | **Product Length (bp)** |
| --- | --- | --- | --- |
| Actin gene | GAGCTGAAAGATTCCGATGC | GCAATGCCTGGGAACATAGT | 178 |
| *AhACO1* | CCATTATTGCTCCTGCACCTA | AAATCTTGGACCCTTCTCACC | 107 |
| *AhACO2* | GAAATGGTGGCAAGCAAAGGC | AGGTAGGAAGATGGCGCAAGA | 94 |
| *AhACS1* | ATGGGAGAAATTAGAGGGAATA | GGTTTGCGAGGATGAAGGTTA | 106 |
| *AhACS2* | ATGAGTCCGTTGCTTGAGAAG | GCATGAAGACCCTGGTGATAT | 88 |
